# A How-To Guide for Mode of Action Analysis of Antimicrobial Peptides

**DOI:** 10.3389/fcimb.2020.540898

**Published:** 2020-10-19

**Authors:** Ann-Britt Schäfer, Michaela Wenzel

**Affiliations:** Division of Chemical Biology, Department of Biology and Biological Engineering, Chalmers University of Technology, Gothenburg, Sweden

**Keywords:** antimicrobial peptides, antibiotics, mode of action, microscopy, membranes

## Abstract

Antimicrobial peptides (AMPs) are a promising alternative to classical antibiotics in the fight against multi-resistant bacteria. They are produced by organisms from all domains of life and constitute a nearly universal defense mechanism against infectious agents. No drug can be approved without information about its mechanism of action. In order to use them in a clinical setting, it is pivotal to understand how AMPs work. While many pore-forming AMPs are well-characterized in model membrane systems, non-pore-forming peptides are often poorly understood. Moreover, there is evidence that pore formation may not happen or not play a role *in vivo*. It is therefore imperative to study how AMPs interact with their targets *in vivo* and consequently kill microorganisms. This has been difficult in the past, since established methods did not provide much mechanistic detail. Especially, methods to study membrane-active compounds have been scarce. Recent advances, in particular in microscopy technology and cell biological labeling techniques, now allow studying mechanisms of AMPs in unprecedented detail. This review gives an overview of available *in vivo* methods to investigate the antibacterial mechanisms of AMPs. In addition to classical mode of action classification assays, we discuss global profiling techniques, such as genomic and proteomic approaches, as well as bacterial cytological profiling and other cell biological assays. We cover approaches to determine the effects of AMPs on cell morphology, outer membrane, cell wall, and inner membrane properties, cellular macromolecules, and protein targets. We particularly expand on methods to examine cytoplasmic membrane parameters, such as composition, thickness, organization, fluidity, potential, and the functionality of membrane-associated processes. This review aims to provide a guide for researchers, who seek a broad overview of the available methodology to study the mechanisms of AMPs in living bacteria.

## Introduction

The discovery of antibiotics has been a major historical milestone. With formerly deadly diseases now being curable with a simple pill, life expectancy, and quality of life increased significantly. The golden age of antibiotics, characterized by the frequent discovery of new lead structures, lasted until the late 1980's. Unfortunately, since the 1990's antibiotic discovery has stagnated while the emergence of multi-resistant bacteria has resulted in untreatable superbugs (Goic-Barisic et al., 2016; Mobarki et al., 2019). The urgent need for new antibiotics prompted a range of interesting alternative strategies and molecules (Spellberg et al., 2015; Silva et al., 2016; Singer et al., 2019).

In order to tackle the antibiotic resistance crisis, novel compounds and novel mechanisms are essential. Bacteria possess a plethora of possible drug targets, yet only few are currently clinically exploited. One promising class of new antibiotic molecules are antimicrobial peptides (AMPs) (Silva et al., 2016; Wang et al., 2016). These omnipresent compounds occur in all domains of life and constitute an effective host defense strategy (Baltzer and Brown, 2011). AMPs are usually defined as up to 100 amino acids long, possess cationic, hydrophobic, and amphipathic properties, and typically target the bacterial cell membrane. Despite these relatively common features, they are a highly diverse class of molecules, both regarding their structures and mechanisms of action (Table 1). The best characterized AMPs are classical pore formers. Different models exist for this mode of action, including the classical barrel stave, the toroidal pore and carpet mechanisms as well as the newer molecular electroporation, sinking raft, and interfacial activity models (Miteva et al., 1999; Pokorny and Almeida, 2004; Chan et al., 2006; Wimley, 2010; Teixeira et al., 2012). Accordingly, mechanisms of AMPs were predominantly investigated using model lipid systems (*in vitro*). However, more and more AMPs are being discovered that have more complex or more subtle interactions with bacterial membranes and do not form pores [e.g., MP196, cWFW, and daptomycin (Wenzel et al., 2014; Scheinpflug et al., 2017; Gray and Wenzel, 2020a)], or do not target membranes at all (Brötz et al., 1998a; Graf et al., 2017; Mishra et al., 2018).

**Table 1:** Overview of different AMPs and antimicrobial proteins and their modes of action.

**Peptide**	**Peptide class**	**Mode of action**	**References**
Daptomycin	Cyclic lipopeptide	Inserts into fluid membrane microdomains that harbor cell envelope synthesis complexes; inhibits cell wall and membrane synthesis by displacing the MurG and PlsX proteins; binds phosphatidylglycerol and undecaprenyl-bound cell wall intermediates	Müller et al., 2016b; Grein et al., 2020
Polymyxin B	Cyclic lipopeptide	Binds to lipopolysaccharides and permeabilizes the outer membrane; integrates into and permeabilizes the cytoplasmic membrane; inhibits respiration	Vaara, 1992; Fu et al., 2019
Surfactin	Cyclic lipopeptide	Membrane permeabilization; local destabilization of membrane packing at low concentrations, detergent-like membrane solubilization at high concentrations	Carrillo et al., 2003; Henry et al., 2011
Bacitracin	Cyclic peptide	Binds to undecaprenylphosphate and leads to inhibition of wall teichoic acid and lipid II synthesis	Ruhr et al., 1971
Gramicidin S	Cyclic beta-sheet peptide	Induces large-scale membrane phase separation and delocalizes peripheral membrane proteins involved in cell division and cell envelope synthesis	Wenzel et al., 2018a
Tyrocidine A	Cyclic beta-sheet peptide	Induces membrane phase separation and forms large transmembrane pores; interferes with DNA-binding proteins and probably induces DNA damage	Ristow et al., 1975; Wenzel et al., 2018a
Theta-defensin	Cyclic beta-sheet peptide	Membrane interaction of theta defensin leads to deregulation of autolytic enzymes and indices autolysis	Wilmes et al., 2014
MP196	RW-rich, cationic antimicrobial peptide (CAMP)	Disturbs membrane organization and delocalizes cytochrome c, MurG, and MinD, resulting in inhibition of respiration, cell wall synthesis, and cell division	Wenzel et al., 2014
cWFW	RW-rich, cyclic CAMP	Separates membrane lipids into fluid and rigid domains, resulting in separation of integral and peripheral membrane proteins in the respective domains, in turn leading to separation of multiprotein complexes	Scheinpflug et al., 2017
LL-37	Alpha-helical CAMP	Membrane disruption by carpet mechanism	Kościuczuk et al., 2012
Aurein 2.1	Alpha-helical CAMP	Forms cation-selective transmembrane pores	Cheng et al., 2009; Wenzel et al., 2015b
Gramicidin A	Alpha-helical peptide	Na^+^/K^+^ channel ionophore	Duax et al., 1996
Magainin	Alpha-helical peptide	Forms a toroidal membrane pore	Ludtke et al., 1996
Alamethicin	Alpha-helical peptaibol	Forms voltage-dependent ion channels	Leitgeb et al., 2007
Vancomycin	Glycopeptide	Inhibits cell wall synthesis by binding to the D-Ala-D-Ala motif of lipid II	Schneider and Sahl, 2010
Nisin	Type A lantibiotic	Binds to lipid II and uses it as a docking molecule to form a transmembrane pore	Breukink et al., 1999
Mersacidin	Type B lantibiotic	Inhibits cell wall synthesis by binding lipid II	Brötz et al., 1998b
hBD3	Beta defensin	Interacts with membranes and displays low affinity for lipid II; probably localizes to sites of active cell wall synthesis and sterically hinders the interaction of protein complexes	Sass et al., 2010
Plectasin	Fungal defensin	Inhibits cell wall synthesis by binding to lipid II	Schneider et al., 2010
Microcin	Lasso peptide	Depolarizes bacterial membranes, stabilizes gel phase in bacterial membrane mimics, RNA polymerase may be an additional target	Delgado et al., 2001; Rintoul et al., 2001, 2015
Valinomycin	Depsipeptide	Potassium carrier ionophore	Duax et al., 1996
Teixobactin	Macrocyclic depsipeptide	Inhibits cell wall synthesis by binding bactoprenol-coupled cell wall precursors	Ling et al., 2015
ADEP	Acyldepsipeptide	Deregulates the ClpP protease, leading to uncontrolled proteolysis of substrates like FtsZ, inhibiting cell division	Brötz-Oesterhelt et al., 2005; Sass et al., 2011
Lysozyme	Antibacterial protein	Lyses the peptidoglycan cell wall by hydrolyzing glycosidic bonds	Aminlari et al., 2014
Actinonin	Peptidomimetic	Inhibits peptide deformylase leading to accumulation of formyl-methionine-capped proteins	Chen et al., 2000

The road to clinical approval can be long and rocky and elucidating the mechanism of action of a new antibiotic can be challenging. Over the last years, a number of methods have been developed, adapted, and refined to investigate the mechanisms of antibiotics in living bacterial cells. This is essential, since the *in vivo* mechanism of a compound can be fundamentally different from its action in artificial models or the molecule may have more than one target, a relatively common feature for AMPs (Sass et al., 2010; Müller et al., 2016b; Wenzel et al., 2019).

In this review, we want to give an overview of the tools available to investigate the *in vivo* mechanisms of both membrane-active AMPs and AMPs with other targets. Thereby, we do not aim to provide an exhaustive list of techniques or detailed summary of all recent technical developments. We rather want to provide a broad handbook for researchers, who are more or less acquainted with mode of action studies, to guide them through a range of possibilities for analyzing the mechanisms of their compounds. While this article is focused on techniques available for studying AMPs, most assays are perfectly suitable to analyze other antibiotic molecules as well. We put special emphasis on analyzing the bacterial cell envelope, but also address other possible targets. Where possible, we selected techniques that can be relatively easily adapted and tried to avoid very specialized niche techniques.

## Compound Localization

Knowing where an antimicrobial compound accumulates in the bacterial cell can give a first hint toward the localization of its target structure. Different labeling approaches have been developed that allow either the detection of compounds in subcellular fractions (e.g., cytosolic, membrane, and cell wall fractions), or the microscopic visualization of antimicrobial molecules. Although being very useful, the chemical labeling of a molecule is bound to change its properties and can change its antimicrobial activity or mechanism of action (Phetsang et al., 2014, 2016; Omardien et al., 2018b; Stone et al., 2018, 2019). Mass spectrometry-based label-free technologies constitute an alternative, yet do not allow visualization of compound localization. The individual advantages and disadvantages of common localization techniques are discussed in the following chapter.

### Radioactive Labeling

Radioactive labeling is the oldest approach to labeling a molecule for following its subcellular distribution and at the same time only minimally invasive to the compound's structure: Radioactive isotopes are generally thought not to affect the chemical properties of a given compound. However, even the mass of an atom can affect its chemical bonds. Thus, mass isotopes can still change the behavior of a labeled molecule (Filiou et al., 2012; Fleming et al., 2014). Radioactive labeling is normally only suitable for antibiotics that can be produced at least semi-synthetically, but it is also possible to obtain radioactively labeled microbially produced antibiotics by growing the producer strain on a radioactive precursor (Atzrodt and Allen, 2011). Radioactive labeling allows very sensitive detection of compounds in subcellular fractions (Perkins and Nieto, 1970; Ishiguro et al., 1981), but it does not allow the visualization of antibiotic localization. Due to these drawbacks and the overall trend to reduce the amount of radioactive material used in research, radioactive labels are typically no longer the method of choice for localizing antimicrobial molecules. However, it may still have its uses in some cases (e.g., for very small molecules that are dwarfed by large fluorescence tags).

### Metal Labeling

A newer approach is metal labeling of antimicrobial compounds. This technique was largely inspired by a ferrocene-containing derivative of the antimalarial drug chloroquine (Biot et al., 2011). Since iron is an electron-dense metal, it should be possible to detect it by electron microscopy. However, iron occurs in bacterial cells in relatively high concentrations, which could lead to a high background signal. This led to the development of a ruthenocene-containing derivative, which was successfully employed to detect the compound in ultrathin sections of malaria parasites (Biot et al., 2012). A similar approach was then employed for a small hexapeptide antibiotic by exchanging the N-terminal amino acid for ruthenocene. This allowed both the visualization of the peptide by electron microscopy and quantification in subcellular fractions by element analysis (Wenzel et al., 2014).

While in this case the activity and mechanism of action of the labeled compound were not notably compromised (Wenzel et al., 2014), it is well-possible that the addition of a metallocene tag will influence the behavior of the compound in one way or another. However, metallocenes are still considerably smaller than common fluorescence labels and thus less likely to severely change the antibiotic properties of a molecule.

Compounds that already contain a residue that can be visualized by electron microscopy (electron-dense metals) or detected by atomic spectroscopy (most elements that do not occur in bacterial cells in high concentrations) can easily be localized without additional labeling (Wenzel et al., 2013). Similarly, AMPs may be visualized with electron microscopy without the need to chemically label them through specific detection with gold-labeled antibodies (Azad et al., 2011). However, this approach requires that the peptide is immunogenic enough to obtain specific antibodies, a property that is normally not desired for antibiotic candidates.

### Fluorescence Labeling

While metal labels only allow the visualization of antimicrobial compounds in fixed cells, fluorescence labels allow live cell imaging of antibiotic attacks on bacterial cells and even co-localization of the antimicrobial molecule with its target. It is a relatively common approach and has aided several mode of action studies so far (Tiyanont et al., 2006; Pogliano et al., 2012; Scheinpflug et al., 2013; Chileveru et al., 2015; Müller et al., 2016b; Omardien et al., 2018b). Most fluorophores have much higher molecular weights than the average antibiotic. Direct labeling with such large moieties may critically influence activity, uptake, and mechanism of action (Katritzky and Narindoshvili, 2009; Müller et al., 2016b; Stone et al., 2019). Even very small fluorescence labels might already compromise antibacterial activity (Scheinpflug et al., 2013). Direct fluorescence labeling approaches can therefore be restricted to larger molecules, which are not severely affected by the addition of a fluorophore (Tiyanont et al., 2006; Chileveru et al., 2015). This generally makes this approach better suited for AMPs than for small molecule antibiotics.

In any case, possible effects of the label on the compound's behavior need to be carefully assessed. This should not be limited to assaying antimicrobial activity alone but also extend to phenotypical characterization to ensure that the compound's mechanism of action has not notably changed. However, the use of fluorescent labels always remains a trade-off between their versatility in live cell microscopy and the possibility that observations made with the labeled compound may not fully translate to its unlabelled original.

An alternative to direct the labeling of AMPs is immunolabelling with fluorescently labeled antibodies. While this approach does not affect the behavior of the compound and still allows microscopic localization studies, it is not suitable for live cell imaging, since it requires permeabilization and chemical fixation of the cells (Choi et al., 2016).

### Label-Free Detection

Label-free detection of antimicrobial compounds by mass spectrometry is an alternative approach that does not have the drawback of compromised activity of labeled compounds. As long as the mass of the molecule of interest is known, it is possible to detect the unlabeled compound in a complex mixture such as cell lysate (Ackermann et al., 1996; Deltombe et al., 2019). This can be used to directly detect and quantify antibiotic concentrations in subcellular fractions. Interestingly, a new approach called 3D imaging cluster Time-of-Flight secondary ion mass spectrometry allowed the label-free detection and mapping of antibiotics in single cells of *Escherichia coli* (Tian et al., 2017). The relatively low spatial resolution of this technique does not allow the visualization of antibiotics to specific cell structures and is therefore not well-suited for mode of action studies yet. However, it gives hope that label-free tracking of antibiotics within bacterial cells might indeed become possible at some point.

However, one limitation that will always remain is that mass spectrometry-based techniques do not allow visualization of antibiotics in living cells. This is an important limitation since more and more evidence is emerging that membrane-targeting AMPs do not uniformly attack the lipid bilayer but instead target specific foci and that their interaction with bacterial membranes can be highly dynamic (Kandaswamy et al., 2013; Rangarajan et al., 2013; Müller et al., 2016b; Rashid et al., 2016). To date, fluorescence labeling remains the only technique that is suitable for capturing these dynamic interactions.

## Finding the Pathway

While the localization of an antimicrobial compound within its target cell helps narrowing down its potential molecular target, it does not give insight into the process or pathway that is actually inhibited and basing hypotheses on localization alone can be misleading. Thus, finding the primarily inhibited pathway is of crucial importance to proceed with detailed mode of action analysis and identifying the molecular target. Classically, this has been done by radioactive precursor incorporation studies, but more recent alternatives include fluorescently labeled precursors and reporter gene fusion.

### Incorporation of Radioactive Precursors

Incorporation experiments with radioactively labeled precursors for the main cellular macromolecules (DNA, RNA, proteins, lipids, peptidoglycan) are very sensitive. While radioactive labeling is commonly sought to be avoided for safety and environmental concerns, it is the only method that allows the detection of macromolecules without altering their chemical structure and thus has the lowest risk of labeling-imposed artifacts. Custom synthesis of radioactively labeled molecules is possible, but commonly used isotopic precursors include [^14^C] glucosamine for peptidoglycan, [^14^C]-thymidine for DNA, [^3^H]-uridine for RNA, L-[^14^C]-isoleucine and [^3^H] glycine for proteins, and [^14^C]-acetate for lipids (Hofmann and Eichenberger, 1998; Ling et al., 2015; Müller et al., 2016b). Some of these labels can be combined in the same sample [e.g., [^14^C] glucosamine and [^3^H] glycine (Molenkamp and Veerkamp, 1976)], yet individual samples are more commonly used). It has to be noted that in order to assess incorporation into macromolecules and not just uptake into cells, samples must be precipitated [e.g., using trichloroacetic acid, prior to measuring radioactivity (Wenzel et al., 2014)]. However, measuring whole cells in parallel is a useful control for cellular uptake, since AMPs often depolarize the cell membrane, which may affect the activity of nutrient uptake systems.

### Fluorescent Labeling of Cellular Macromolecules

An alternative to radioactive labeling of metabolites is constituted by fluorescent labeling. A range of fluorescent molecules have been developed that can be used to cover some of the major metabolic pathways of bacterial cells. The simplest example for this is probably the expression of a fluorescent protein, such as green-fluorescent protein (GFP), from a housekeeping or inducible promoter, which allows monitoring of active protein synthesis in living bacterial cells (Gray et al., 2019). A more direct approach is metabolic labeling of nascent peptide chains with the amino acid analog L-homopropargylglycine (L-HPG), followed by fluorescent labeling of this molecule with Alexa-594 by click chemistry (Stempler et al., 2017; Gray et al., 2019). Several similar probes have been described and specific reporters for certain posttranslational modifications are available as well (Grammel and Hang, 2013).

Incorporation of cell wall material can be monitored by fluorescent D-amino acids or sortase-mediated incorporation of fluorescently labeled lipid II (Nelson et al., 2010; Kuru et al., 2012, 2015; Hsu et al., 2017). Similarly, fluorescently labeled glycans can be incorporated into the Gram-negative or mycobacterial outer membrane (Siegrist et al., 2015). These techniques are described in detail under 6.2 Cell wall and 6.1 Outer membrane, respectively.

Fluorescent nucleotide analogs that can be incorporated into DNA or RNA have been developed for eukaryotic cells, but their suitability for bacterial cells has not yet been explored (Grammel and Hang, 2013).

Fluorescent labeling of metabolic precursors is superior to radioactive labeling in terms of safety and official regulations, can be visualized in living bacterial cells, and in some cases allows further analysis of the labeled macromolecules, for example by affinity purification of the tag followed by mass spectrometry (Grammel and Hang, 2013). However, it is an inherent limitation of chemically modified precursors that they may not behave exactly as the unlabeled molecule. This can be due to the size of the fluorescent tags, which are often larger than the precursor itself, or simply to changing the physicochemical properties of the target molecule (Siegrist et al., 2015).

### Reporter Gene Fusions

A simple alternative to precursor incorporation studies are reporter gene fusions. Bacteria react to stress in a highly specific manner. So much so that the stress response can be used as a diagnostic tool to identify antibiotic mechanisms of action (see also four Profiling approaches) (Bandow et al., 2003). Based on this, specific reporters can be selected for mapping the inhibited pathway, analogous to precursor incorporation experiments (Urban et al., 2007). To this end, either the gene of interest or only its promoter, is fused to a gene encoding a reporter protein, whose expression can be visualized by calorimetric, fluorescent, or luminescent measurements. The most common reporter genes encode firefly luciferase or beta-galactosidase, but fluorescent proteins like GFP are also possible.

The main advantage of this method is that it does not need radioactive labeling, does not produce artifacts by chemical modification of precursors, and does not require any major or unusual equipment. However, the choice of reporter genes or promoters requires solid knowledge of bacterial stress responses and a new set of strains has to be constructed for each organism of interest. This also limits it to model organisms that are genetically accessible. Thus, reporter gene approaches for antibiotic mode of action analysis are most common in the standard Gram-positive and Gram-negative model organisms *Bacillus subtilis* and *E. coli* (Table 2) (Bianchi, 1999; Hutter et al., 2004a; Urban et al., 2007; Wenzel et al., 2014). However, reporter gene studies in general are common tools in many organisms, including pathogens like *S. aureus*, and also strains that were not developed as antibiotic mode of action analysis tool can prove useful as such (Mesak et al., 2008; Chanda et al., 2009; Mondal et al., 2010; Dengler and McCallum, 2016; Bojer et al., 2017).

**Table 2:** Examples of reporter gene fusions commonly used to identify antibiotic mechanisms.

**Promoter**	**Fusion**	**Species**	**Reporter for**	**References**
*bmrC*	Luciferase	*B. subtilis*	Inhibition of translation	Wenzel et al., 2014
*fabHB*	Luciferase	*B. subtilis*	Inhibition of fatty acid synthesis	Hutter et al., 2004a
*glpD*	Luciferase	*B. subtilis*	Inhibition of fatty acid synthesis	Hutter et al., 2004a
*Held*	Luciferase	*B. subtilis*	Inhibition of transcription	Wenzel et al., 2014
*liaI*	Luciferase	*B. subtilis*	Inhibition of cell wall synthesis	Wenzel et al., 2014
*yheI*	Luciferase	*B. subtilis*	Inhibition of protein synthesis	Urban et al., 2007
*yorB*	Luciferase	*B. subtilis*	DNA damage	Wenzel et al., 2014
*ypbG*	Luciferase	*B. subtilis*	Inhibition of cell wall synthesis	Hutter et al., 2004a
*ypuA*	Luciferase	*B. subtilis*	Cell wall stress	Hutter et al., 2004a
*yrzI*	Luciferase	*B. subtilis*	Inhibition of protein synthesis	Hutter et al., 2004a
*yvgS*	Luciferase	*B. subtilis*	Inhibition of RNA synthesis	Urban et al., 2007
*drp35*	β-galactosidase	*S. aureus*	Inhibition of cell wall synthesis	Mondal et al., 2010
*dnaK*	β-galactosidase	*E. coli*	Protein misfolding	Bianchi, 1999
*Ibp*	β-galactosidase	*E. coli*	Protein misfolding	Bianchi, 1999
*P3rpoH*	β-galactosidase	*E. coli*	Extracytoplasmic stress	Bianchi, 1999
*degP)*	β-alactosidase	*E. coli*	Extracytoplasmic stress	Bianchi, 1999

New reporter gene tools are constantly developed and refined. For example, a modified luciferase reporter assay reporting on cell wall synthesis and DNA integrity enables antibiotic mode of action analyses and screening of new drugs against *Mycobacterium tuberculosis* (Naran et al., 2016). Efforts to enable cost-efficient high through put screenings with reporter gene fusions have recently resulted in the development of a phenomics screening platform containing an *E. coli* reporter gene library enabling large-scale gene expression studies in a cost- and time-efficient manner (French et al., 2018).

## Profiling Approaches

While precursor incorporation and reporter gene experiments offer a great way to quickly identify the affected pathway, they only scratch the surface. A much deeper understanding is made possible by -omics approaches that allow global profiling on genomic, transcriptomic, proteomic, and metabolomic level. These techniques are very useful for generating hypotheses about antibiotic mechanisms but can rarely stand all alone. While they offer a large amount of information, complex datasets also require a significant amount of time for analysis and may be difficult to interpret for antimicrobial compounds with multiple or pleiotropic effects, which is often the case for AMPs.

-omics approaches are certainly not a must in mode of action analysis of “typical” AMPs that impair membrane integrity, but they allow an unmatched combination of breadth and depth of physiological insight and can be extremely valuable for compounds with unknown/unusual mechanisms. The amount of technical variations, especially in mass spectrometry-based proteomics, is immense and we do not remotely attempt to cover them all. In the next chapter we want to present selected techniques that have a well-established standing in antibiotic mode of action studies.

### Genomic Profiling

Genomic-driven approaches have gained much attention in antibiotic drug discovery, mainly as sources for new antibiotic targets (Miesel et al., 2003; Freiberg et al., 2005). However, genomic approaches can also aid in identifying antibiotic targets. They can be roughly divided into two groups, screening existing libraries and generating new mutant libraries.

For both *E. coli* (Baba et al., 2006) and *B. subtilis* (Koo et al., 2017), commercially available mutant collections exist that comprise deletion strains of each non-essential gene. These collections can be screened against hypersensitivity to or resistance against an antibiotic of interest to discover potential resistance factors or target candidates, respectively. This has been systematically approached by Tamae et al. and Liu et al., resulting in sensitivity patterns for close to 30 different antibiotic compounds (Tamae et al., 2008; Liu et al., 2010), which can be used as a reference for studying novel drug candidates (Tran et al., 2011; Kang et al., 2012). One obvious limitation of this approach is that it does not include essential genes, which are commonly thought to be the most suitable antibiotic targets. Recently, CRISPR knock-down libraries covering essential genes have been established for both *B. subtilis* and *E. coli* (Peters et al., 2016; Guo et al., 2018; Wang et al., 2018). Both libraries have been made commercially available. While they have not yet been used in antibiotic mode of action studies, they complement the genomic toolbox available for such approaches. However, when working with such mutant libraries, care has to be taken that relevant strains are independently confirmed and that updated annotations of the mutated open reading frames are taken into account (Baba et al., 2006; Yamamoto et al., 2009; Aedo et al., 2019).

The second approach aims at generating resistant mutants that may reveal the molecular target of an antimicrobial compound. This can for example be done by characterizing spontaneous resistant mutants generated under lower antibiotic pressure or repeated passaging of incrementally resistant colonies on rising antibiotic concentrations (Leejae et al., 2013; Puertolas-Balint et al., 2020). The latter is prone to result in accumulation of different mutations, complicating analysis and interpretation, and is likely to result in unstable mutants due to the combined fitness costs of multiple mutations (Leejae et al., 2013). An alternative approach is the generation of mutants by chemical or transposon mutagenesis followed by selecting for antibiotic-resistant colonies. Thereby, transposon mutagenesis is the easier option, since it allows rapid identification of the insertion locus by sequencing from the transposon sequence (Santiago et al., 2018). In contrast, spontaneous mutants and chemical mutagenesis require whole genome sequencing to map individual mutations. However, transposon mutagenesis precludes analyzing targets encoded by essential genes, while spontaneous and chemically induced mutations do not necessarily result in loss of function.

### Transcriptomic Profiling

While genomic approaches map the level of antibiotic sensitivity, transcriptomic and proteomic approaches map the stress response profiles of bacteria to antibiotic stress, which are diagnostic for the individual compound's mechanism of action and can aid target identification (Bandow et al., 2003; Bandow and Hecker, 2007; Wenzel and Bandow, 2011). While microarrays have been the predominant technique for transcriptomic profiling for a long time, RNA sequencing is now the method of choice in most cases, since it is more sensitive, does not rely on hybridization probes, and is becoming more and more affordable (Hutter et al., 2004b; Gilad et al., 2009; O'Rourke et al., 2020). Many studies have successfully used transcriptomic profiling to aid mode of action analysis (Briffotaux et al., 2019; O'Rourke et al., 2020) and its uses have been extensively reviewed elsewhere (Freiberg et al., 2004; Wecke and Mascher, 2011). However, it should be noted that parameters for stress response profiling, be it transcriptomic or proteomic experiments, must be chosen with care. Thus, sublethal antibiotic concentrations and short treatment times should be used in order to achieve the best possible acute stress response (Wenzel and Bandow, 2011; Raatschen and Bandow, 2012).

### Proteomic Profiling

While transcriptomic profiling is well-suited to monitor antibiotic stress responses, proteomic profiling can provide additional information on posttranslational modifications and regulation mechanisms, such as proteolysis. Metabolic labeling, either radioactively for gel-based proteomics or with stable isotopes for mass spectrometry, allows highly sensitive pulse and pulse-chase experiments for monitoring acute stress responses at a given time point.

Gel-based proteomics by two-dimensional polyacrylamide gel electrophoresis (2D-PAGE) is a proteomic approach that has been extensively employed in stress response profiling and antibiotic mode of action research (Bandow et al., 2003; Mostertz et al., 2004; Wecke et al., 2009). To this end, newly synthesized proteins are radioactively pulse labeled with L-[^35^S] methionine and, after a crude protein extraction, separated according to their isoelectric point and molecular weight. Protein expression is then densitrometrically quantified from autoradiographs of dried gels and compared to an untreated control to acquire regulation factors. Upregulated proteins, referred to as marker proteins, are identified by mass spectrometry. These proteins reflect the acute stress response of the bacterial cells to the given stress condition and are indicative of the antibiotic mechanism of action. A reference compendium with protein expression profiles of over 100 antimicrobial compounds has been established to aid mode of action analysis of new drug candidates (Bandow et al., 2003; Wenzel et al., 2011, 2012, 2013, 2014, 2015b; Raatschen et al., 2013; Stepanek et al., 2016a,b; Müller et al., 2016b; Scheinpflug et al., 2017; Saising et al., 2018; Meier et al., 2019; Wüllner et al., 2019). Radioactive 2D-PAGE should not be confused with two-dimensional difference gel electrophoresis (2D-DIGE), which also compares protein expression profiles by densitometric quantification against a control sample, but is a fluorescent sample multiplexing and not a metabolic labeling technique (Minden, 2012).

Radioactive 2D-PAGE has been proven very robust and potent in the field but has its practical limitations mainly in terms of equipment needed, handling of and regulations around radioactive samples, and relatively low throughput. Moreover, it is not suitable for membrane proteomics, which may be particularly interesting for AMPs. Gel-free proteomics does not suffer from these limitations and may be more accessible to many researchers while providing a similar outcome for mechanistic studies. The gel-free counterpart of radioactive 2D-PAGE, in terms of metabolic labeling, is stable isotope labeling by amino acids in cell culture (SILAC). Here, one culture (e.g., the untreated control), is grown in a medium containing a ^13^C-labeled amino acid, while another culture, e.g., the antibiotic-treated sample, is grown on normal medium. This allows pooling of the samples and quantification in the same run and can also be done as a pulse experiment to selectively label only newly synthesized proteins (Snider et al., 2019). Instead of using mass-labeled amino acids, labeling can also be achieved with other sources of heavy nitrogen, such as ammonium (Dreisbach et al., 2008; Wenzel et al., 2014). As with radioactive 2D-PAGE and 2D-DIGE, SILAC should not be confused with iTRAQ (isobaric tag for relative and absolute quantitation), which is not a metabolic labeling but a multiplexing technique (Unwin, 2010).

Recently, label-free approaches have been heavily employed for mode of action analysis of antimicrobials (Müller et al., 2016a; Stepanek et al., 2016b; Gao et al., 2017; Kang et al., 2019; Yuan et al., 2020). However, these detect differences in protein levels and not newly synthesized proteins, rather giving insight into successfully completed stress adaptation rather than acute stress response. Label-free proteomics is most powerful when employed together with a technique that detects the acute stress response, such as radioactive 2D-PAGE or transcriptomics (Darby et al., 2014; Müller et al., 2016a; Stepanek et al., 2016b), but can also effectively aid mode of action analysis by itself (Opoku-Temeng et al., 2019; Ajdidi et al., 2020).

### Metabolomic Profiling

Metabolomics is a comparatively young -omics technique that has not been extensively employed for mechanistic antibiotic studies yet. In contrast to genomics, transcriptomics, and proteomics (Bandow et al., 2003; Tamae et al., 2008; Liu et al., 2010; O'Rourke et al., 2020), there are no large comparative metabolomic studies on antibiotic stress yet. However, bacterial metabolomics has been employed for a variety of applications including identification of new antibiotics and characterizing resistance mechanisms (Gao and Xu, 2015; Wu et al., 2015; Li et al., 2019) and is emerging as a tool in mode of action studies (Wang et al., 2019; Liu et al., 2020). Metabolomics can be employed as a more detailed approach to find the affected metabolic pathway and thus constitute a sensitive alternative to radioactive and fluorescent precursor incorporation studies, or as an in-depth analysis of antibiotic effects on the bacterial metabolism. However, interpreting large metabolomic datasets requires profound knowledge of the metabolic networks in the respective organism.

## Cell Morphology

An alternative or additional starting point to mode of action analysis can be cell morphology. Many antibiotics, in particular AMPs, cause distinct defects in cell shape, size, or integrity that can be observed by both light and electron microscopic techniques (Friedrich et al., 2000). While examination of cell morphology alone does normally not identify a mechanism of action, it provides a good basis for further phenotypical analysis and the combination of rather simple morphological assays can be used to effectively map mechanistic classes (Nonejuie et al., 2013).

### Electron Microscopy

A classical method to examine bacterial morphology is transmission electron microscopy (TEM). TEM has been frequently employed to study antibiotic effects on bacterial cells, since it offers unique insight into bacterial ultrastructures (Friedrich et al., 2000; Sass et al., 2011; Nicolas et al., 2019; Vazquez-Muñoz et al., 2019). Sample preparation for TEM involves chemical fixation, dehydration, and contrasting with metal stains, followed by embedding in resin and ultrathin sectioning. One limitation of TEM comes into effect when working with rod-shaped bacteria like *E. coli* or *B. subtilis*: due to the random orientation of bacteria in the resin the majority of cells is cross-sectioned, which makes it difficult to assess antibiotic-induced phenotypes. This limitation was recently overcome by a flat embedding approach, where bacteria are aligned on an agarose film prior to embedding, resulting in mostly longitudinally sectioned cells (Wenzel et al., 2019).

Scanning electron microscopy (SEM) is an electron microscopy technique, which allows inspection of the bacterial cell surface in great detail but does not allow imaging intracellular structures. SEM has been successfully employed for antibiotic mode of action studies and is particularly interesting for AMPs, which often cause cell surface defects (Zweytick et al., 2014; Yang et al., 2017; Nicolas et al., 2019). For SEM, cell samples are also fixed, dehydrated, and contrasted with a metal stain, but normally not cut into sections (Kaláb et al., 2008). Environmental SEM (ESEM) omits the need for critical point drying and enables imaging of hydrated samples (Collins et al., 1993).

### Atomic Force Microscopy

Another form of microscopy that detects surface changes is atomic force microscopy (AFM). AFM makes use of a small needle, the cantilever, to scan over a sample and record the force it encounters when interacting with the sample surface. This allows the generation of height profiles, measuring of cell surface stiffness, and detection of cellular content leakage (Dorobantu and Gray, 2010; Neethirajan and DiCicco, 2014). AFM is often chosen as a method to examine changes in bacterial biofilms (Dorobantu and Gray, 2010) and is recently gaining more attention as a tool to examine bacterial cell morphology for antibiotic mode of action analysis (Meincken et al., 2005; Mularski et al., 2016). Moreover, antibiotic-induced morphological changes measured by AFM can aid identifying antibiotic-resistant strains (Ierardi et al., 2017) distinguishing between persister and resister phenotypes (Uzoechi and Abu-Lail, 2020).

### Bacterial Cytological Profiling

A relatively new tool for fast mode of action analysis is bacterial cytological profiling (BCP). This fluorescence light microscopy-based technique is a combination of different staining techniques that give a quick overview of major cellular components (Nonejuie et al., 2013; Lamsa et al., 2016). Using principal component analysis, antibiotics can be grouped into classes and new compounds can be rapidly assigned to a mechanistic group. An expansion of this method, called rapid inhibition profiling (RIP), allows mapping the target pathway of compounds with novel targets. This method makes use of proteolytic degradation of a potential antibiotic target protein to generate a reference cytological profile, against which new antibiotic candidates can be compared. This allows a reliable identification of new mechanisms of action (Lamsa et al., 2016; Peters et al., 2018).

Originally starting with essentially a membrane and DNA stain, BCP has been further refined and expanded over the years and has aided a number of mode of action studies (Pogliano et al., 2012; Nonejuie et al., 2016; Mohammad et al., 2017; Htoo et al., 2019). Nowadays, it may include a variety of fluorescent dyes and protein fusions in addition to or in place of the original membrane and DNA dyes (Araujo-Bazan et al., 2016; Müller et al., 2016b; Omardien et al., 2018a,b; Saeloh et al., 2018; Wenzel et al., 2018a). Since a clear definition of bacterial cytological profiling is missing, the term may be used for a distinct combination of two or three dyes for high throughput pathway mapping as well as for comprehensive bacterial cell biology studies. To get a first glance at cell morphology changes, the combination of a red membrane dye (typically FM5-95, FM4-64, or Nile red), a blue DNA dye (typically DAPI), and phase contrast microscopy has been proven useful (Nonejuie et al., 2013; Saeloh et al., 2018; Wenzel et al., 2018a). These dyes are easy to handle, do not require specialized fluorescence filters, and can be combined with a GFP fusion of interest, for example the cell division protein FtsZ (Araujo-Bazan et al., 2016). However, there is a broad palette of fluorescence dyes and protein fusions available that report on various cellular functions and components and have been successfully employed in antibiotic mode of action studies. We will describe a number of such specialized fluorescence reporters in the following chapter.

## Cellular Components

Once the target pathway or structure has been mapped, the next step in mode of action analysis is a detailed assessment of the mechanism of action and identification of the target structure. While a specific drug-target interaction is normally always confirmed with purified components *in vitro*, antibiotics may have different of additional targets in living cells (Müller et al., 2016b; Wenzel et al., 2019). A number of *in vivo* methods is available to study the effects of antimicrobial compounds on living bacteria. In the following, we will describe assays that can be used to assess the effects of AMPs and other antibacterial molecules on the major components of a bacterial cell.

### Outer Membrane

The lipopolysaccharide-rich outer membrane is highly impermeable and the first line of defense of Gram-negative bacteria. It is a major intrinsic antibiotic resistance factor and the main reason why Gram-negative bacteria are much more resilient to antibiotic attacks than Gram-positive bacteria. Antibiotics that impair this permeability barrier are urgently needed (Silhavy et al., 2006) and some AMPs have been shown to target the outer membrane, most prominently the polymyxins (Vaara, 1992).

Outer membrane permeability can be assayed with fluorescent dyes such as 1-N-phenylnaphthylamine (NPN) and 1,8-anilino-1-napthalenesulfonicacid (ANS). NPN and ANS have a relatively week fluorescent signal in aqueous solution but exhibit strong fluorescence in hydrophobic environments like lipid membranes. These dyes do not penetrate the outer membrane and thus do not stain intact cells. Upon outer membrane permeabilization however they can bind to membrane phospholipids leading to an increased fluorescence signal (Loh et al., 1984; Schved et al., 1994; Gravel et al., 2017). ANS is sensitive to charge neutralization, leaving NPN as the dye of choice for polycationic compounds like many AMPs (Loh et al., 1984).

Similarly, outer membrane permeability can be assayed by testing the sensitivity to small molecule antibiotics that normally do not penetrate the outer membrane, but do have a target in Gram-negative cells (Heesterbeek et al., 2019). In contrast to fluorescence dyes, this method does not allow quantification of outer membrane permeabilization. However, using antibiotics of different molecular weight, the outer membrane pore size can be estimated. Antibiotics that can be used for this are for example rhodomyrtone (442.54 g/mol), vancomycin (1449.3 g/mol), and nisin (3354.07 g/mol).

Another method to assess the integrity of the outer membrane is AFM, which reports on cell surface stiffness, which is a direct measure for outer membrane or, in Gram-positive bacteria, cell wall integrity and can therefore be employed to measure the effects of outer membrane-targeting compounds.

Outer membrane proteins can also be used as a proxy to determine outer membrane integrity. One simple way to do this, is to isolate outer membrane fractions and perform a Western blot analysis of outer membrane proteins (Rojas et al., 2018). Alternatively, proteins can be identified and quantified by mass spectrometry. In addition to these outer membrane integrity assays, there are also fluorescence labeling approaches available to visualize glycans on the outer membrane (Backus et al., 2011; Siegrist et al., 2015).

### Cell Wall

**Figure 1:**
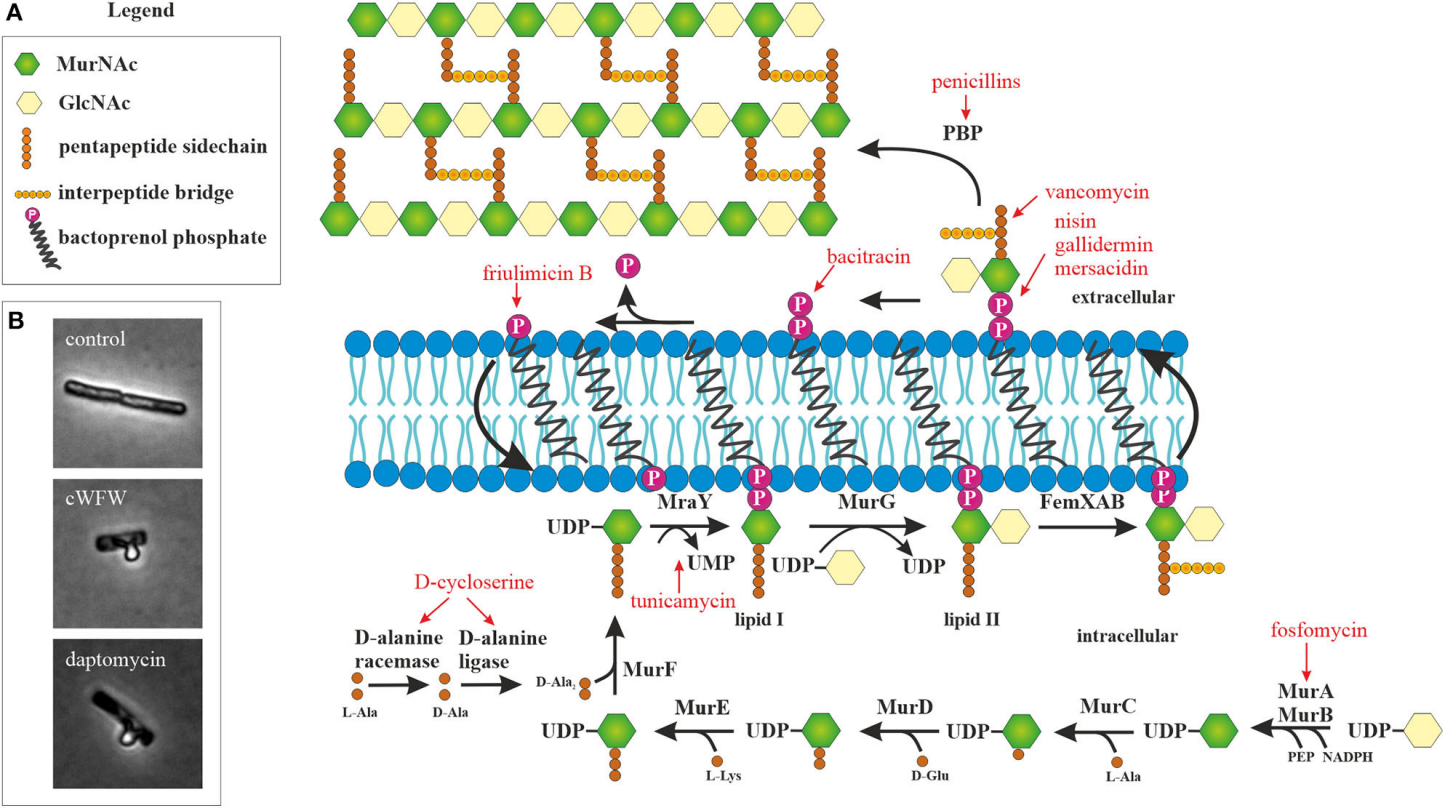
Peptidoglycan synthesis as antibiotic target. **(A)** Overview of peptidoglycan synthesis in *S. aureus* and antibiotics targeting this pathway (modified from Schneider and Sahl, 2010). Peptidoglycan synthesis is a common target of peptide antibiotics. With the exception of tunicamycin and fosfomycin, all antibiotics in this figure are peptide-based. **(B)** Acetic acid/methanol fixation of *B. subtilis*. Inhibition of cell wall synthesis leads to extrusion of the protoplast through breaches in the peptidoglycan layer.

The next barrier after the outer membrane, and the first barrier in Gram-positive bacteria, is constituted by the peptidoglycan cell wall. With few exceptions of cell wall-less bacteria, this structure is essential for bacterial survival. It does not only protect the cell from mechanical stress, but also prevents it from bursting due to turgor pressure. Cell wall synthesis is a complex and highly coordinated process that takes place partly in the cytosol and partly in the cytoplasmic membrane (Figure 1). Together with the bacterial ribosome, the cell wall synthesis machinery is the most successful antibiotic target in the clinic and at the same time the most common target of AMPs after the cytoplasmic membrane (Yeaman and Yount, 2003; Schneider and Sahl, 2010). Moreover, recent studies suggest that the membrane interaction of AMPs severely disturbs the synthesis of the peptidoglycan precursor lipid II (Sass et al., 2010; Wenzel et al., 2014; Müller et al., 2016b).

Due to its utmost clinical relevance and the relatively frequent discovery of new cell wall-active agents, a broad method spectrum is available to analyze the effects of compounds on this pathway, in particular its interaction with lipid II. This includes various reporter gene assays, *in vitro* lipid II synthesis, lipid II binding visualized by thin layer chromatography, and detection of accumulated lipid II by high performance liquid chromatography (HPLC), to name only a few standard techniques (Brötz et al., 1998a; Schneider et al., 2009, 2010; Schneider and Sahl, 2010; Ling et al., 2015). Taking HPLC-based detection of cell wall components a step further, recent studies have succeeded to refine the isolation of cell wall peptidoglycan and detect glycan strain length and crosslinking, allowing detailed analysis of cell wall peptidoglycan composition (Desmarais et al., 2014, 2015; Montón Silva et al., 2018; More et al., 2019).

A fast assay that can also be used to screen for cell wall synthesis inhibitors is the AmpC reporter assay (Sun et al., 2002). In this assay, the beta-lactamase gene *ampC* and its regulator *ampR* from *Citrobacter freundii* are cloned into *E coli*. This system senses accumulated cell wall degradation products and soluble cell wall precursors and is induced upon inhibition of peptidoglycan synthesis by a broad spectrum of antibiotics, not only by beta-lactams. Using an optical density-based beta-lactamase survival assay, beta-lactamase expression in response to the antimicrobial compound of interest can be monitored. However, it has to be noted that this assay does not respond to every cell wall synthesis inhibitor tested, thus not providing complete coverage (Sun et al., 2002).

A simple microscopic assay to assess whether incorporation of cell wall precursors is inhibited is the acetic acid/methanol fixation (Schneider et al., 2010; Wenzel et al., 2012). In Gram-positive bacteria, this treatment leads to extrusion of the protoplast through holes in the cell wall matrix (Figure 1B). The peptidoglycan layer is a dynamic structure that is constantly remodeled to accommodate cell growth and division. To this end, autolytic enzymes constantly break down the cell wall at specific sites to accommodate incorporation of new cell wall material. If these holes are not filled because lipid II synthesis is inhibited, a much higher proportion of cells with membrane extrusions are observed in the fixation assay. However, deregulation of autolytic enzymes may have similar effects.

**Figure 2:**
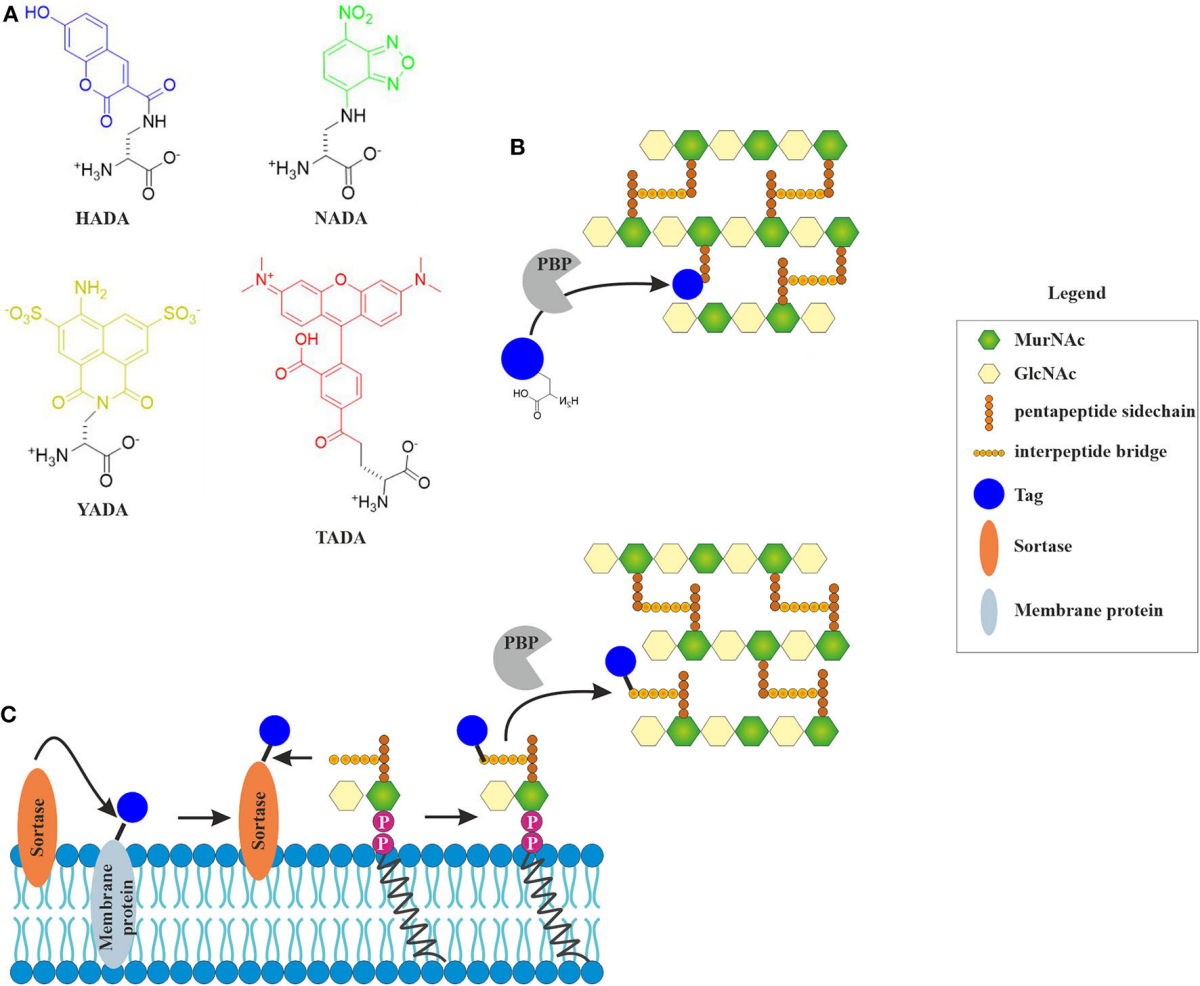
Incorporation of fluorescent cell wall labels. **(A)** Structures of different fluorescently labeled amino acids (FDAAs) with different spectral properties (HADA: blue, NADA: green, YADA: yellow, TADA: red) (Kuru et al., 2015; Hsu et al., 2017). **(B)** FDAAs mimic the peptide side chain of the peptidoglycan precursor and are incorporated into the cell wall by bacterial enzymes (modified from Hsu et al., 2017). **(C)** Incorporation of fluorescent tags into the peptidoglycan layer by sortase-mediated labeling. The fluorescent tag is coupled to a membrane protein through a linker that contains a signal peptide sequence that is cleaved by the sortase enzyme. The free tag can then bind lipid II through a nucleophilic attack. This results in a tagged lipid II, which is incorporated into the cell wall by penicillin-binding proteins (Hendrickx et al., 2011).

Fluorescently labeled D-amino acids (FDAAs) have been a major breakthrough in the field, since they for the first time allowed the direct visualization of active incorporation of cell wall precursors into living bacterial cells under the microscope (Kuru et al., 2015; Hsu et al., 2017). FDAAs mimic the D-amino acids in the peptide side chain of the peptidoglycan precursor and are incorporated into the cell wall by penicillin-binding proteins (HADA) or L-D-transpeptidases (NADA) (Figure 2) (Montón Silva et al., 2018). Incorporation of FDAAs into the cell wall does not appear to be toxic for bacteria. Since their original discovery, new FDAAs have been designed in different fluorescent colors, making them readily available for co-localization experiments (Hsu et al., 2017). However, the different FDAAs have their advantages and disadvantages. For example, HADA is sensitive to photobleaching, while NADA requires higher concentrations to achieve a satisfactory fluorescence signal, and TDL, a red-fluorescing FDAA, only weakly stains *E. coli* (Kuru et al., 2015).

**Figure 3:**
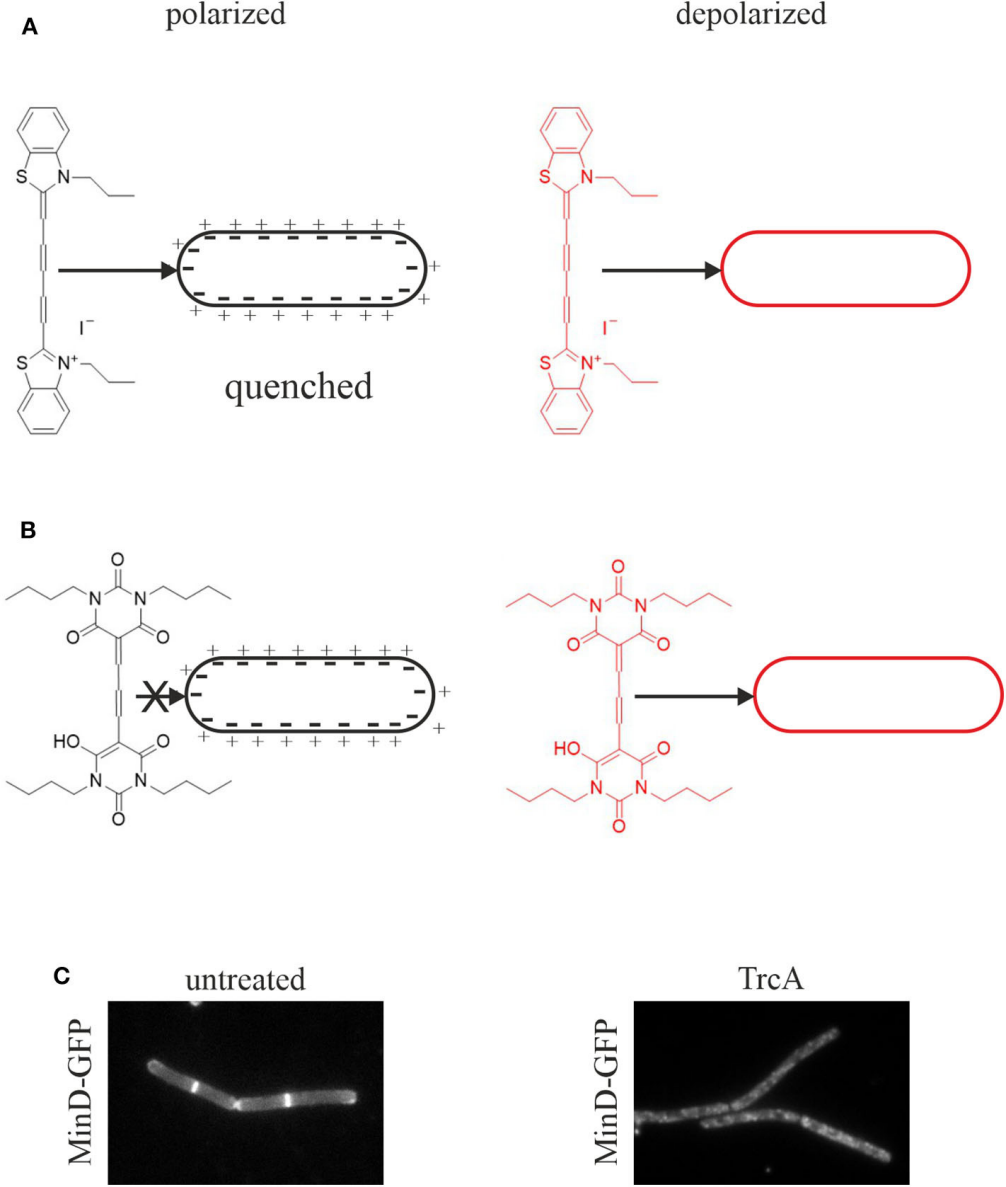
Assays for membrane depolarization. **(A)** Membrane depolarization assay with DisC_3_(5). This self-quenching dye inserts into polarized membranes and is released upon depolarization leading to an increased fluorescent signal. **(B)** Membrane potential assay with DiBAC4(3). This dye does not insert into polarized membranes and is only self-quenching at very high concentrations. Upon depolarization it inserts into the membrane resulting in an increased fluorescence signal (te Winkel et al., 2016). **(C)** Depolarization assay with the GFP-MinD reporter strain. MinD normally localizes at the cell poles and the cell division plane. Membrane depolarization, here by tyrocidine A, leads to disturbance of this regular pattern and a spotty GFP signal over the whole cell membrane and partial dislocation of the protein into the cytosol (Strahl and Hamoen, 2010).

A different way of visualizing the effects of antibiotics on cell wall precursors, it sortase-mediated fluorescence labeling of lipid II, which generally works well for Gram-positive species (Nelson et al., 2010). While incorporation efficiency of FDAAs is likely inhibited by antibiotics, sortase-mediated labeling will be largely unaffected (Sugimoto et al., 2017). Sortase is a membrane-bound protease that cleaves a signal peptide sequence off transmembrane proteins. This can be used to cleave a fluorescence tag, which can be biotin, azide, or a fluorescent chromophore (Nelson et al., 2010), from a transmembrane protein. This tag can then react with lipid II, producing a labeled version of the precursor on the membrane surface (Figure 3). If an antibiotic interferes with cell wall synthesis, this will lead to mislocalization or clustering of the labeled molecule.

Another way to visualize cell wall synthesis components is the labeling of antibiotics with the fluorescence tag 4,4-difluoro-4-bora-3a,4a-diaza-s-indacene (BODIPY). BODIPY is a very common fluorescence tag that can be easily conjugated with a number of biomolecules, including antibiotics that inhibit cell wall synthesis. Vancomycin-BODIPY (Van-FL) and penicillin-BODIPY (bocillin) are commercially available. Van-FL binds to the D-Ala-D-Ala motif of lipid II and has been successfully used to visualize lipid II (Pogliano et al., 2012; Schirner et al., 2015). Bocillin binds to penicillin-binding proteins (PBPs) and can be used for visualizing the localization of these proteins or for competition experiments with other PBP inhibitors. Since bocillin does not recognize all PBPs, BODIPY fusions to other PBP inhibitors can be employed to distinguish between PBP subpopulations (Stone et al., 2018).

### Cytoplasmic Membrane

The cytoplasmic membrane is the target of the majority of AMPs. A plethora of biophysical techniques are available to assay the parameters of model membranes and such assays have been excessively used to characterize the membrane interactions of AMPs *in vitro*. However, the true complexity of bacterial membranes cannot be mimicked, since not only the composition, but also the physicochemical properties vary drastically from species to species, between different growth conditions, between media, and growth phases. It has become apparent that model membrane studies are not enough to truly describe the complex nature of AMP-membrane interactions, the most prominent example being daptomycin (Pogliano et al., 2012; Müller et al., 2016b; Gray and Wenzel, 2020a; Grein et al., 2020). This realization together with the growing interest in microbial membrane architecture (Jones et al., 2001; Lopez and Kolter, 2010; Barák and Muchová, 2013; Bramkamp and Lopez, 2015; Strahl and Errington, 2017) has prompted the development of a variety of *in vivo* techniques to analyze membrane physiology in living bacteria. The amount of available techniques would go beyond the scope of this review, but we will describe a number of relatively easily accessible techniques that are well-suited for mode of action analysis of AMPs.

#### Membrane Composition

Bacterial membranes are complex mixtures of roughly equal parts of lipids and proteins. The lipid composition of bacterial membranes is far from static and varies depending on a variety of factors. Thus, bacteria readily adapt their membrane composition under antibiotic stress (Fränzel et al., 2010; Saeloh et al., 2018). The membrane composition of bacteria can be analyzed in different ways. Head group composition can be easily analyzed using thin layer chromatography (Pogmore et al., 2018), a technique that does not require expensive instrumentation or access to mass spectrometry facilities. Fatty acid composition can be measured by gas chromatography (Saeloh et al., 2018). Lipidomics can also be performed by mass spectrometry allowing sensitive detection of lipid species, detection of head groups and fatty acids, and fingerprinting [e.g., to identify bacterial species according to their lipid profile (Fränzel et al., 2010; Rezanka et al., 2015; Hewelt-Belka et al., 2016)].

Antimicrobial compounds may bind to a specific lipid species. One example for this is daptomycin, which binds to phosphatidylglycerol lipids and prefers fluid membrane environments (Hachmann et al., 2011; Müller et al., 2016b). Mutant analysis is a powerful tool to investigate such preferences. For example, *B. subtilis* mutants lacking specific head groups, such as phosphatidylethanolamine or lysyl-phosphatidylglycerol and mutants with altered membrane fluidity have been established and some of them have been used for antibiotic mode of action analysis (Salzberg and Helmann, 2008; Mercier et al., 2012; Saeloh et al., 2018; Gohrbandt et al., 2019). However, some lipid species are essential. For example, in contrast to *E. coli* phosphatidylglycerol-free *B. subtilis* cells are not viable. In such cases depletion strains can be used (Murray and Koh, 2014).

#### General Membrane Dyes

Several fluorescence dyes are available to visualize cell membranes under the microscope. Some of them are specific for a certain membrane parameter, while others are rather unselective, general membrane dyes. The latter ones are a good tool for simple bacterial cytological profiling experiments and co-localization with membrane proteins or cell wall labels. For bacteria, mainly red and green fluorescence dyes are used (Table 3). MitoTracker Green (MTG) is a very bright green membrane dye. It provides an excellent signal-to-noise ratio and very good contrast for high resolution techniques like structured illumination microscopy (SIM) (Saeloh et al., 2018). However, prolonged exposure to MTG is toxic for bacteria and leads to artifacts. MTG also stains the forespore membrane in *B subtilis* (Schneider et al., 2007).

**Table 3:** Selection of fluorescence dyes applied in mode of action experiments.

**Dye**	**Reports on**	**Example study on bacteria**
NPN	Outer membrane permeability	Loh et al., 1984
ANS	Outer membrane permeability	Schved et al., 1994
Bocillin	Penicillin-binding proteins	Pogliano et al., 2012
Van-FL	Lipid II	Pogliano et al., 2012
HADA	Sites of active cell wall synthesis	Schirner et al., 2015
NADA	Sites of active cell wall synthesis	Montón Silva et al., 2018
Mitotracker green (MTG)	General membrane dye	Saeloh et al., 2018
Nile red	General membrane dye	Saeloh et al., 2018
FM5-95	General membrane dye	Müller et al., 2016b
FM4-64	General membrane dye	Pogliano et al., 2012
NAO	Negatively charged phospholipids	Pogmore et al., 2018
DiIC12	Fluid membrane microdomains	Wenzel et al., 2018b
DPH	Membrane fluidity	Bessa et al., 2018
Laurdan	Membrane fluidity	Wenzel et al., 2018b
DiSC3(5)	Membrane potential	te Winkel et al., 2016
DiBAC4(3)	Membrane potential	te Winkel et al., 2016
APG-2	Potassium flux	Saeloh et al., 2018
Propidium iodide	Pores	Jiang et al., 2019
Sytox green	Pores	Barns and Weisshaar, 2013
BCECF	pH	Strahl and Hamoen, 2010
Resazurin	Respiratory chain activity	Saeloh et al., 2018
INT	Respiratory chain activity	Dutton et al., 1983
CTC	Respiratory chain activity	Rodriguez et al., 1992
CellRox	Reactive oxygen species	Wenzel et al., 2013
Oxyburst green	Reactive oxygen species	Surewaard and Kubes, 2017
DCFH-DA	Reactive oxygen species	Arakha et al., 2015
DAPI	DNA	Nonejuie et al., 2013
SYTO9	DNA	Krychowiak et al., 2018
SYTO RNAselect	RNA	Bakshi et al., 2014

*This list is not exhaustive and only includes dyes that have been used for antibiotic mode of action studies in bacteria. Note that some of these dyes have several derivatives covering different wavelengths (e.g., SYTO dyes) or having different membrane-penetrating properties (e.g., APG dyes)*.

Nile red is a relatively photostable bright red-fluorescent dye. It is easy to handle and also provides a good contrast. However, the dye readily adsorbs to glass cover slips resulting in a high background. Coating the cover slips with poly-L-dopamine resolves this issue and allows using the dye for SIM (te Winkel et al., 2016; Saeloh et al., 2018). Nile red is often used for co-localization studies with GFP-labeled proteins. Yet, its brightness and broad excitation and emission make it prone to bleeding into other channels (Ohsaki et al., 2010). Like MTG, Nile red is toxic upon prolonged exposure and additionally enhances phototoxicity, making it unsuitable for timelapse microscopy.

Alternatives to Nile red are the red-fluorescent general membrane dyes FM5-95 and FM4-64. They are less bright than Nile red and strongly adsorb to glass cover slips. While dopamine coating prevents this well enough for normal microscopy, these dyes do normally not provide sufficient contrast for SIM. They are better suited for co-localization studies due to lower bleed through and are not toxic for bacterial cells, making them the dyes of choice for timelapse experiments (Pogliano et al., 2012; Müller et al., 2016b; Wenzel et al., 2018a).

All of these dyes, like most dyes and proteins, will transition into the fluid phase upon phase separation (Müller et al., 2016b; Scheinpflug et al., 2017; Saeloh et al., 2018; Gohrbandt et al., 2019), making them a robust tool to assess membrane phase separation induced by membrane-active AMPs (Scheinpflug et al., 2017).

#### Membrane Pores

AMPs are typically thought to form pores in bacterial membranes. While this is certainly the case for some AMPs, there are many others that affect the membrane in different ways (Wenzel et al., 2014, 2018a; Müller et al., 2016b; Scheinpflug et al., 2017). The common pore models of AMP action are mainly derived from *in vitro* assays and confirmation of such results *in vivo* is pivotal. Several tools are available for this purpose (Table 3).

A simple method to assess the formation of large pores is to monitor efflux of proteins, for example intracellular GFP (Yoneyama et al., 2009). This efflux can also be followed using timelapse microscopy visualizing the attack of AMPs on bacterial cells in a time-resolved manner (Barns and Weisshaar, 2016).

One of the most common pore assays is propidium iodide staining, in combination with a SYTO9 counterstain also marketed under the name BacLight Live/Dead assay. Both dyes bind to DNA, but only SYTO9 can cross intact membranes resulting in green-fluorescent cells. If the membrane is perforated, and given that pores are big enough, propidium iodide can enter resulting in red-fluorescent cells. This assay can be used microscopically, but is also very popular for fluorescence-activated cell sorting (FACS) (Freire et al., 2015; Patra et al., 2015).

A similar stain is Sytox green, a green-fluorescent dye that can only enter cells through membrane pores. Sytox green has been successfully employed for real-time monitoring of pore formation by AMPs (Barns and Weisshaar, 2013; Rangarajan et al., 2013). Sytox is also available in other colors (e.g., red and deep red), allowing combination with other dyes. Similarly, the membrane-permeable SYTO dyes, which are often used as counterstains, are available in variants covering the full spectrum of visible light.

Leakage of smaller intracellular molecules like amino acids or nucleotides can be measured by HPLC (Wenzel et al., 2015b; Ye et al., 2018). Smaller, ion-conducting pores can be monitored with different techniques. Commonly, potassium-selective electrodes are employed (Wenzel et al., 2012; Münch et al., 2014). The advantage of measuring potassium with an ion-selective electrode is that it can be done in a time-resolved manner. However, AMPs may adsorb to the electrode surface causing measurement artifacts or even damaging the electrode. Another limitation is the ion selectivity itself, since a compound may as well be an ionophore selective for another ion. This can be assayed with total ion analysis, also referred to as ionomics, which is typically measured by inductively coupled atomic emission spectroscopy (ICP-AES) or ICP mass spectrometry (ICP-MS) (Baxter, 2010; Wenzel et al., 2014; Müller et al., 2016b).

Another method to measure ions are ion-sensitive dyes, such as Asante potassium green (APG-2) or Asante sodium (natrium) green (ANG-2). APG-2 has been successfully employed for measuring potassium efflux from *B. subtilis* cells treated with antibiotics (Saeloh et al., 2018). Both APG-2 and ANG-2 are available as membrane-permeable acetoxymethyl (AM) ester for intracellular measurements and as membrane-impermeable tetramethylammonium (TMA) salt for extracytoplasmic measurements. Similarly, proton concentrations can be measured intra- and extracellularly using the pH-sensitive dye 2′,7′-bis-(2-carboxyethyl)-5-(and-6)-carboxyfluorescein (BCECF), which also comes as AM and TMA version (Strahl and Hamoen, 2010).

The combination of cytosolic GFP leakage, fluorescent pore stains, leakage of small cellular molecules, and measuring ion permeability allows insight into pore size.

#### Membrane Potential

Pore formation, regardless of large or small, will result in membrane depolarization. However, depolarization can be achieved without the noticeable presence of pores. Thus, impaired respiration may diminish the proton gradient and/or small transient ion currents may contribute to depolarization. Such effects may not be captured in pore assays. Therefore, the membrane potential should always be measured in addition to leakage assays. Two fluorescent dyes are well-established for membrane potential measurements in bacteria, 3,3′-dipropylthiadicarbocyanine iodide (DiSC3(5)) and bis-(1,3-dibutylbarbituric acid) trimethine oxonol (DiBAC4(3)) (Figure 3).

DiSC3(5) is a self-quenching dye that inserts into polarized membranes (Figure 3A). It is released upon depolarization leading to an increased fluorescent signal in spectroscopic assays (te Winkel et al., 2016). DiSC3(5) is a sensitive dye and captures small potential changes as well as transient depolarization with accuracy. Using the potassium ionophore valinomycin, the measurements can be calibrated allowing quantitative information about the membrane potential (te Winkel et al., 2016). DiSC3(5) can be used microscopically allowing single cell analysis and assessing heterogeneity in the cell population. It is a far-red dye and thus compatible with GFP fusions and most other dyes commonly used in bacterial cell biology. However, prolonged exposure to DiSC3(5) is toxic for bacterial cells preventing its use in timelapse microscopy (te Winkel et al., 2016). While this dye does not bind to glass cover slips, it does interact with polydimethylsiloxane, a common material for microfluidic devices (te Winkel et al., 2016).

DiBAC4(3) is an alternative to DiSC3(5) with a slightly different mechanism (Figure 3B). This dye does not insert into polarized membranes and is only self-quenching at very high concentrations. It can only insert into depolarized membranes, which results in an increased cellular fluorescence signal due to the locally higher dye concentration (te Winkel et al., 2016). It is used for both membrane potential measurements and cell viability assays (Jepras et al., 1997). DiBAC4(3) is not toxic to bacteria during prolonged incubation and is therefore suitable for timelapse microscopy (te Winkel et al., 2016). However, the dye binds strongly to glass surfaces, which can be prevented by poly-L-dopamine coating (te Winkel et al., 2016).

A simple and quick experiment to check for membrane depolarization is the MinD delocalization assay. MinD is a protein involved in the regulation of cell division site positioning in rod-shaped bacteria. It binds to the cell membrane with an amphipathic alpha-helix motif and localizes at the cell poles and the cell division site. This localization is severely disturbed upon depolarization (Strahl and Hamoen, 2010). In *B. subtilis*, depolarization leads to a spotty localization pattern and partial dissociation of the protein from the membrane into the cytosol (Figure 3C). In *E. coli*, MinD oscillates from pole to pole, which can be easily observed by timelapse microscopy. Dissipation of the membrane potential abolishes this oscillation (Strahl and Hamoen, 2010). The cell division protein FtsA and the cell shape determining protein MreB are also delocalized upon membrane depolarization, FtsA being released from the membrane into the cytosol and MreB forming clusters at the cell membrane (Strahl and Hamoen, 2010). However, MinD is the most popular proxy for dissipation of the membrane potential and has been used in a number of studies (Chimerel et al., 2012; Eun et al., 2012; Foss et al., 2013; Wenzel et al., 2015a).

In *E. coli*, it is also possible to measure the membrane potential directly by patch clamp of giant spheroplasts generated from cells treated with the cell septation inhibitor cephalexin (Sun et al., 2014; Kikuchi et al., 2015). Cephalexin selectively binds to PBP4, a penicillin-binding protein involved in septation (Kocaoglu and Carlson, 2015). This results in a septum-free, elongated phenotype suitable for generating giant spheroplasts for patch clamp.

It has to be noted that microscopy slides coated with poly-lysine, which are sometimes used to immobilize cells, already cause depolarization of bacterial cells. Such slides should never be used for bacterial studies, neither for depolarization assays nor any other experiment on live cells, since membrane depolarization causes a plethora of pleiotropic effects that cannot be distinguished from antibiotic effects (Strahl and Hamoen, 2010).

#### Membrane Fluidity

Membrane fluidity has recently emerged to play a central role in the mechanism of action of antibiotics and AMPs (Hachmann et al., 2009; Strahl et al., 2014; Müller et al., 2016b; Scheinpflug et al., 2017; Omardien et al., 2018b; Wenzel et al., 2018a). Membrane fluidity is essentially defined as the viscosity of the cell membrane and can be affected by a number of factors, such as membrane composition, proteins, or temperature. It is difficult to define the overall fluidity of a biological membrane and there is no method to measure this directly. Instead, the complex factors contributing to membrane fluidity are best described by a number of assays that report on each of these factors separately to obtain a differentiated picture of the biological membrane system.

Membrane composition is a crucial factor for fluidity that bacteria can adapt according to the environmental conditions (Zhang and Rock, 2008). In *B. subtilis*, which has more than 90% branched-chain fatty acids, membrane fluidity is mainly controlled by the ratio of *iso* and *anteiso*-branched-chain fatty acids. Fast stress adaptation is additionally achieved by the degree of fatty acid desaturation (Beranová et al., 2008; Kingston et al., 2011). These ratios can be assayed by lipid analysis as described earlier.

Membrane fluidity crucially affects the movement of proteins within the lipid bilayer. The diffusion of membrane proteins can therefore be used as a measure for overall membrane fluidity. Thus, fluorescence recovery after photobleaching (FRAP) can be employed to assay this membrane parameter. FRAP is a fluorescence microcopy method that uses laser-based photobleaching of a small area of a cell, usually a cell pole. Recovery of the fluorescence signal by diffusion of fluorophores into the previously bleached area can be observed by timelapse microscopy. When this experiment is performed with a fluorescently labeled membrane protein, the fluorescence recovery rate gives a measure for membrane fluidity (Devkota and Pilon, 2018). FRAP is one of the most direct techniques to monitor membrane fluidity. However, in bacteria the photobleached region is quite large compared to the cell size, which does not allow precise measurements of specific membrane domains. Thus, it can only be used as a measure for general membrane fluidity over the whole cell membrane.

Fluorescence dyes are another alternative to measure membrane fluidity. 1,6-diphenyl-1,3,5-hexatriene (DPH) is a hydrophobic trans-polyene that inserts into the membrane bilayer and orientates itself parallel to the fatty acid chains (Los and Murata, 2004). DPH is a fluorescence polarization probe. Its rotational mobility directly depends on membrane fluidity, allowing DPH polarization to be used as a direct measure of this membrane parameter (Fox and Delohery, 1987). DPH has been employed to assess membrane fluidity in antibiotic-treated bacteria including *E. coli, B. subtilis*, and *Staphylococcus aureus* (Bessa et al., 2018; Gohrbandt et al., 2019). DPH delivers robust spectroscopic data but cannot be visualized under the microscope.

**Figure 4:**
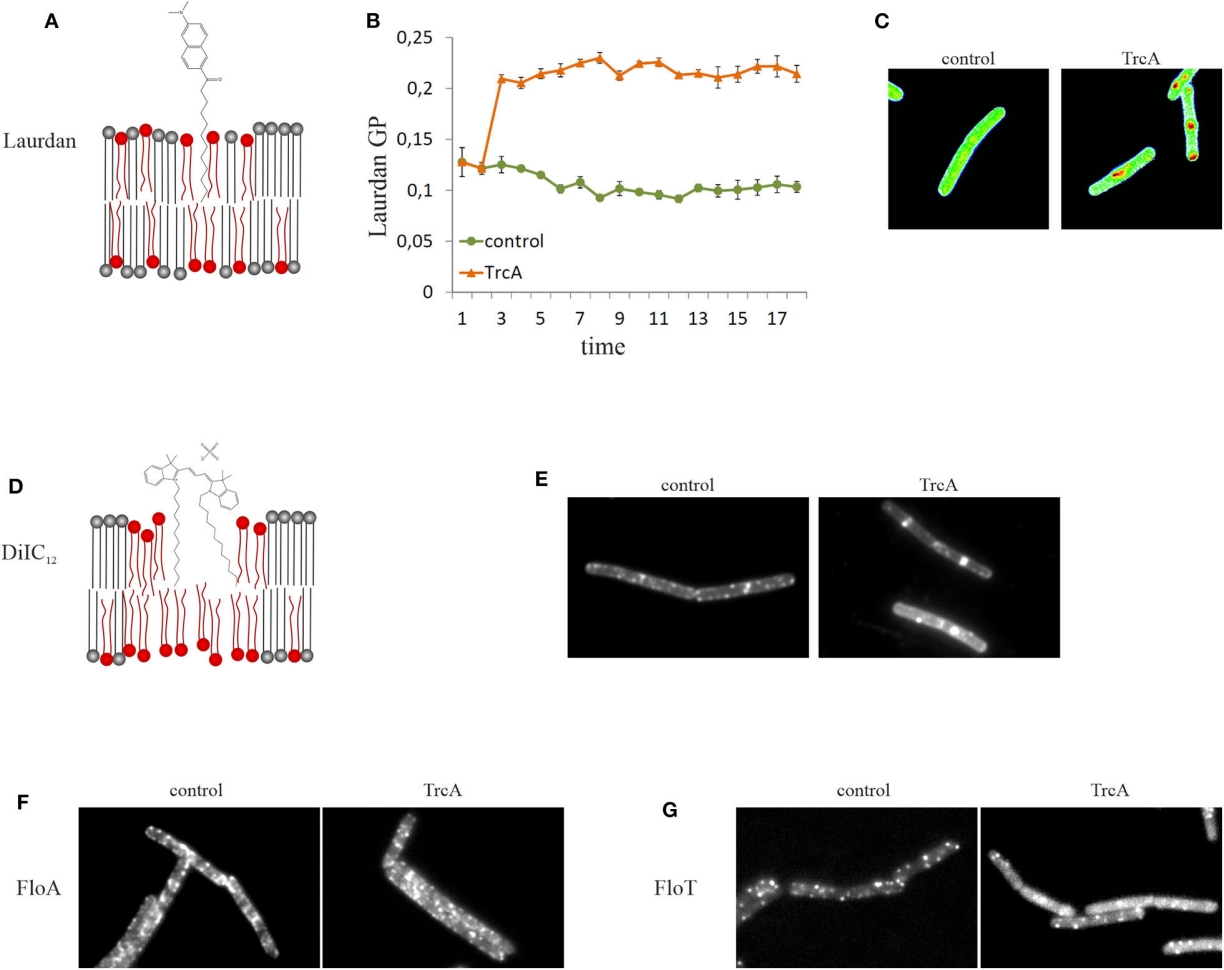
Tools for microscopic examination of membrane domains of different fluidity. **(A)** Laurdan is a fluorescent membrane dye that exhibits a fluidity-dependent fluorescence emission shift (schematic not to scale). **(B)** Laurdan can be used in spectroscopic assays allowing recording of the kinetics of overall membrane fluidity changes. The cyclic beta-sheet peptide tyrocidine A causes rapid membrane rigidification. **(C)** Laurdan can also be visualized under the microscope and a GP scale can be created using ImageJ. Tyrocidine-induced membrane domains appear much more fluid (red) than the rest membrane (green). **(D)** DiIC12 preferentially inserts into fluid membrane environments and is therefore well-suited to detect RIFs and other fluid membrane domains (schematic not to scale). **(E)** Treatment with tyrocidine A disturbs the distribution of RIFs and induces their fusion into large fluid domains. **(F,G)** Flotillins are reporters for rigid membrane domains (lipid rafts). Delocalization of FloA **(F)** and FloT **(G)** by tyrocidine A indicates that this peptide affects membrane domains.

An alternative to DPH is the fluidity-sensitive membrane dye 2-dimethylamino-6-lauroylnaphtalene, commonly known as laurdan. Detection of membrane fluidity by laurdan is based on a fluorescence emission shift depending on the amount of water molecules that surround the probe. Thus, laurdan is not a direct measure of membrane viscosity, but indicates lipid head group spreading and fatty acid chain mobility, which are crucial factors for the fluidity of biological membranes (Parasassi and Gratton, 1995; Sanchez et al., 2007). Laurdan is excited at 350 nm and emission is recorded at 460 and 500 nm. Calculating the generalized polarization [GP = (I_460_ – I_500_)/(I_460_ + I_500_)] gives a measure for membrane fluidity. Laurdan GP can be measured both spectroscopically and microscopically (Figure 4). A detailed protocol for laurdan-based fluidity measurements in bacteria has been published recently (Wenzel et al., 2018b). Both DPH and laurdan can also be used in model membrane systems, which can be a useful as a control to discern direct from indirect antibiotic effects (Tyteca et al., 2003; Saeloh et al., 2018; Wenzel et al., 2018b).

Another parameter that influences membrane fluidity is membrane thickness, with fluid membranes being thinner than rigid bilayers (Reddy et al., 2012; Karabadzhak et al., 2018). AMPs can induce both, membrane thinning and thickening (Grage et al., 2016). Biophysical methods to measure membrane thickness of model membranes include solid state ^2^H-NMR and grazing incidence small X-ray scattering (GISAXS) (Grage et al., 2016). However, methods to measure membrane thickness *in vivo* are scarce. It has been proposed that pore-forming alpha-helical peptides of different length can be employed as “molecular rulers” to measure membrane thickness *in vivo* (Grau-Campistany et al., 2015). While this is a very interesting approach, its suitability for determining antibiotic-induced changes in membrane thickness remains to be evaluated. However, it can be expected that the combined effects of the ruler peptides and the antimicrobial molecule of interest may affect the results. It would also not be suited for any type of pore-forming compound, which would interfere with the readout of the assay, being either cell viability or membrane permeability (Grau-Campistany et al., 2015).

Membrane thickness can be qualitatively assessed *in vivo* with the fluorescence dye 1,1′-didodecyl-3,3,3′,3′-tetramethylindocarbocyanine perchlorate (DiIC12). However, DiIC12 is rather a fluid membrane domain dye than an accurate reporter for membrane thickness (Figure 4).

#### Membrane Domains

Bacterial membranes are heterogenous lipid mixtures and contain different types of lipid domains. DiIC12 stains fluid membrane domains based on its preference for thinner membrane regions, which is determined by its short 12C hydrocarbon tail (Baumgart et al., 2007; Zhao et al., 2013). Rod-shaped bacteria that grow through lateral expansion of the cell wall possess fluid membrane domains that harbor the lateral cell wall synthesis machinery. These domains are termed regions of increased fluidity (RIF). In *B. subtilis* and *E. coli* DiIC12 displays a clear preference for RIFs (Figure 4) (Strahl et al., 2014; Oswald et al., 2016). In the cocci *S. aureus* and *Streptococcus pneumoniae*, which do not possess RIFs as such, DiIC12 still produces a heterogenous membrane stain suggesting that these bacteria have fluid lipid domains as well (Saeloh et al., 2018; Gray and Wenzel, 2020a). It has to be noted that at least in *B. subtilis* RIFs can only be observed in exponentially growing cells. In stationary phase cells, DiIC12 staining results in a smooth membrane stain (Wenzel et al., 2018a). A detailed protocol for this fluid lipid domain stain has been published recently (Wenzel et al., 2018b).

While analogous dyes with longer hydrocarbon tails exist (e.g., DiIC18), they do not seem to stain rigid membrane domains (Strahl et al., 2014). Currently, no dye exists that displays affinity for thicker or more rigid membrane domains in bacteria. However, larger rigid domains can be visualized with laurdan (Scheinpflug et al., 2017). Also, the absence or strong reduction of the signal of a fluorescent membrane stain or protein may indicate a gel-phase membrane domain. However, one has to keep in mind that gel-phase domains are thought not to exist in bacterial membranes under normal conditions and only occur through intense membrane stress (Wenzel et al., 2018a).

Certain membrane proteins can be used as proxies for membrane domains of different fluidity. Thus, the lipid II synthase MurG and the phospholipid synthase PlsX localize in RIFs in exponentially growing *B. subtilis* (Müller et al., 2016b). MreB also co-localizes with RIFs both in *B. subtilis* and *E. coli*, but less strictly than MurG and PlsX (Strahl et al., 2014; Oswald et al., 2016). In contrast, flotillins are proteins that are thought to be associated with and stabilize lipid rafts, may be used as proxies for rigid membrane domains (Lopez and Kolter, 2010; Bach and Bramkamp, 2013; Wagner et al., 2020). In *B. subtilis* known flotillins are the integral membrane protein FloA and the peripheral FloT (Figure 4) (Dempwolff et al., 2012). However, a recent study has shown evidence that these proteins may be associated with fluid rather than rigid domains (Zielińska et al., 2020), challenging the established paradigm and questioning the use of flotillins as raft reporters in bacteria.

Many membrane-active compounds including several AMPs affect the distribution of membrane domains. RIFs seem to be very sensitive to this, which makes sense since membrane areas with higher fluidity better accommodate molecules than less fluid membranes. A common phenotype appears to be the fusion of RIFs leading to accumulation of lipid dye in these antibiotic-induced fluid domains (Omardien et al., 2016; Saeloh et al., 2018; Wenzel et al., 2018a). This does not only affect DiIC12, but most membrane dyes including MTG, Nile red, FM5-95, laurdan, and DiSC(3)5, as well as many membrane proteins (Müller et al., 2016b; te Winkel et al., 2016; Saeloh et al., 2018; Wenzel et al., 2018a). However, a similar phenotype can be observed for membrane invaginations. Typically, invaginations are too small to be seen by wide-field microscopy, but the double or multiple membrane layers will result in a locally increased fluorophore concentration. To distinguish between a fluid domain and an invagination, structured illumination microscopy (SIM) can be employed (Mercier et al., 2013; Saeloh et al., 2018; Wenzel et al., 2018a). Alternatively, a GFP fusion to the *B. subtilis* AtpA protein can be used (Johnson et al., 2004). This ATP synthase subunit does not accumulate in fluid membrane domains, but due to its uniform distribution over the membrane does accumulate in membrane invaginations (Saeloh et al., 2018; Wenzel et al., 2018a).

Apart from membrane domains of different fluidity, lipid domains characterized by specific head group species have been proposed, most prominently cardiolipin domains. These domains have been visualized with the positively charged membrane dye nonyl acridine orange (NAO), which stains negatively charged phospholipids (Mileykovskaya and Dowhan, 2009). However, this well-established concept has recently been challenged, when Pogmore et al. showed that these domains are in fact artifacts caused by stress inflicted through the staining procedure. In fact, domains appeared in a *B. subtilis* strain fully devoid of cardiolipin, when the standard staining protocol was used. In contrast, the wild type strain stained with a stress-free protocol showed no accumulation of the dye in domains (Pogmore et al., 2018).

#### Membrane-Bound Processes

One crucial component of bacterial membranes is often neglected in mode of action studies of AMPs, namely membrane-bound proteins, which make up about half of the mass of the cytoplasmic membrane. While many AMPs disrupt membrane integrity at high peptide to lipid ratios, their minimal inhibitory and bactericidal concentrations are often far below the concentrations needed for efficient membrane permeabilization (Gray and Wenzel, 2020a). It is therefore likely that effects on essential membrane-bound processes caused by perturbations of membrane fluidity or architecture are responsible for growth inhibition and cell death at these concentrations. Moreover, more and more membrane-active molecules are found that do not kill bacteria by membrane permeabilization but by interfering with the coordination of membrane-bound processes (Sass et al., 2010; Wenzel et al., 2014, 2018a; Wilmes et al., 2014; Jahn et al., 2015; Scheinpflug et al., 2017; Saeloh et al., 2018).

One extensive method to assess the effect of antibiotics on membrane proteins is what could be called extended bacterial cytological profiling. BCP is often used as a relative high-throughput assay for mode of action classification and antimicrobial susceptibility testing that relies on the combination of typically two to four key reporters (Nonejuie et al., 2013; Araujo-Bazan et al., 2016; Lamsa et al., 2016; Quach et al., 2016). However, it can also be used in more extensive studies by examining a broad panel of GFP fusions, often in combination with some of the dyes discussed above (Müller et al., 2016b; Omardien et al., 2018a; Wenzel et al., 2018a). GFP fusions can be employed for this approach in two ways: to determine which processes are affected by an antibiotic and as reporters for certain membrane parameters [e.g., MinD for membrane depolarization (Strahl and Hamoen, 2010; te Winkel et al., 2016)]. Table 4 shows a selection of GFP fusions commonly employed for antibiotic mode of action studies in *B. subtilis*.

**Table 4:** Proteins commonly used for bacterial cytological profiling and their localization in *B. subtilis* (Müller et al., 2016b; Saeloh et al., 2018; Wenzel et al., 2018a).

**Protein**	**Protein function**	**Reporter for**	**Localization**
HbsU	Regulation of nucleoid compaction	Chromosome compaction	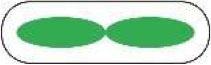
ParB	Chromosome positioning before septation	DNA replication	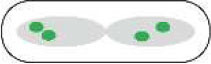
PolC	Alpha-subunit of the DNA polymerase III	DNA replication	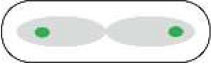
DnaN	Beta-subunit of the DNA polymerase III	DNA replication and repair	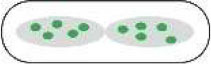
RecA	Homologous recombination and DNA repair	DNA damage	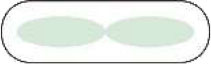
RpoC	Beta-subunit of the RNA polymerase	RNA synthesis	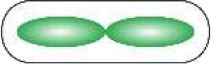
RpsB	Ribosomal protein	Protein synthesis	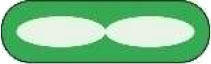
PgsA	Biosynthesis of phospholipids	Phospholipid synthesis	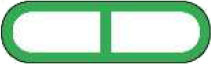
PlsX	Phospholipid synthase	Phospholipid synthesis	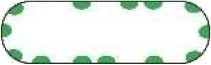
FloA	Flotillin	Membrane domains	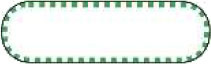
FloT	Flotillin	Membrane domains	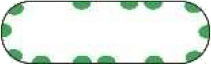
SdhA	Succinate dehydrogenase	Membrane-bound energy generation	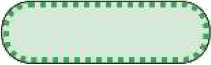
AtpA	ATP synthase	Membrane invagination	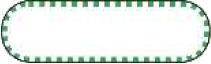
DivIVA	Cell division regulation	Cell division	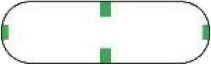
MinD	Cell division regulation	Cell division	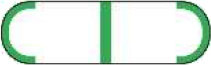
FtsA	Membrane anchor of the cell division protein FtsZ	Cell division	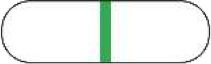
FtsZ	Major cell division protein, forms the Z-ring	Cell division	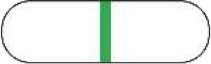
MreB	Cell shape-determining protein	Cytoskeleton	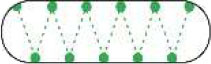
MreC	Cell shape-determining protein	Cytoskeleton	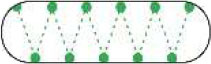
MreD	Cell shape-determining protein	Cytoskeleton	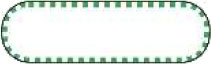
MurG	Lipid II synthase	Cell wall synthesis	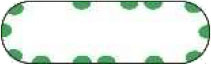
MraY	Lipid I synthase	Cell wall synthesis	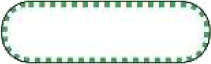
PBP2B	Penicillin-binding protein 2B	Cell wall synthesis	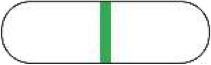
PonA	Penicillin-binding protein 1A/1B	Cell wall synthesis	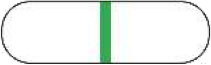
FtsW	Peptidoglycan glycosyltransferase	Cell wall synthesis	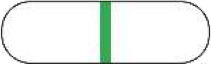

The localization of a protein is crucial for its correct function making BCP is a reliable assay to map affected processes. However, it does not yield information about the degree of inhibition. Therefore, it may be necessary to additionally test the functionality of a membrane-bound process of interest. The most commonly affected pathways are cell wall synthesis (assays described above) and the respiratory chain.

Activity of the respiratory chain can be measured in different ways. One option is the dye resazurin, which can be reduced to the differently colored resorufin. The probe is an indicator of an active respiratory chain since the reduction to resorufin is proportional to aerobic respiration (González-Pinzón et al., 2012). Resazurin can also be used in isolated inverted membrane vesicles and is compatible with both calorimetric and fluorescence detection. Alternatively, tetrazolium dyes can be used. For example, the colorless 5-cyano-2,3-ditolyl tetrazolium chloride (CTC) can be reduced to a bright red-fluorescing CTC-formazan through an active electron transport chain (Rodriguez et al., 1992). A similar dye that is employed for this purpose is 2-(p-iodophenyl)-3-(p-nitrophenyl)-5-phenyl tetrazolium chloride (INT) (Dutton et al., 1983). Both CTC and INT are suitable for fluorescence spectroscopy and microscopy.

Inhibition of the respiratory chain typically leads to depletion of cellular ATP levels. This can be measured with commercially available chemiluminescence assays (Wenzel et al., 2014; Scheinpflug et al., 2017), or detected by HPLC or mass spectrometry (Dudley and Bond, 2014; Ye et al., 2018).

Several other membrane-bound processes may be inhibited by AMPs, including cell division, membrane and teichoic acid synthesis, protein secretion, nutrient uptake systems, virulence, or motility. Listing possible assays for each membrane-bound process would go beyond the scope of this article but it is good to be aware that disruption of membrane lipids can have a plethora of effects on membrane proteins, from almost universal protein delocalization to very specific effects on a small number of proteins (Müller et al., 2016b; Wenzel et al., 2018a; Gray and Wenzel, 2020b).

### Proteins

The direct interaction of antibiotics with specific protein targets is typically confirmed by *in vitro* binding and activity assays. However, this requires that a candidate protein is already known, which is normally only the case for derivatives of known antimicrobial compounds. Finding a candidate protein target from scratch can be difficult and in the following we will describe a selection of methods that help identify candidate target proteins.

**Figure 5:**
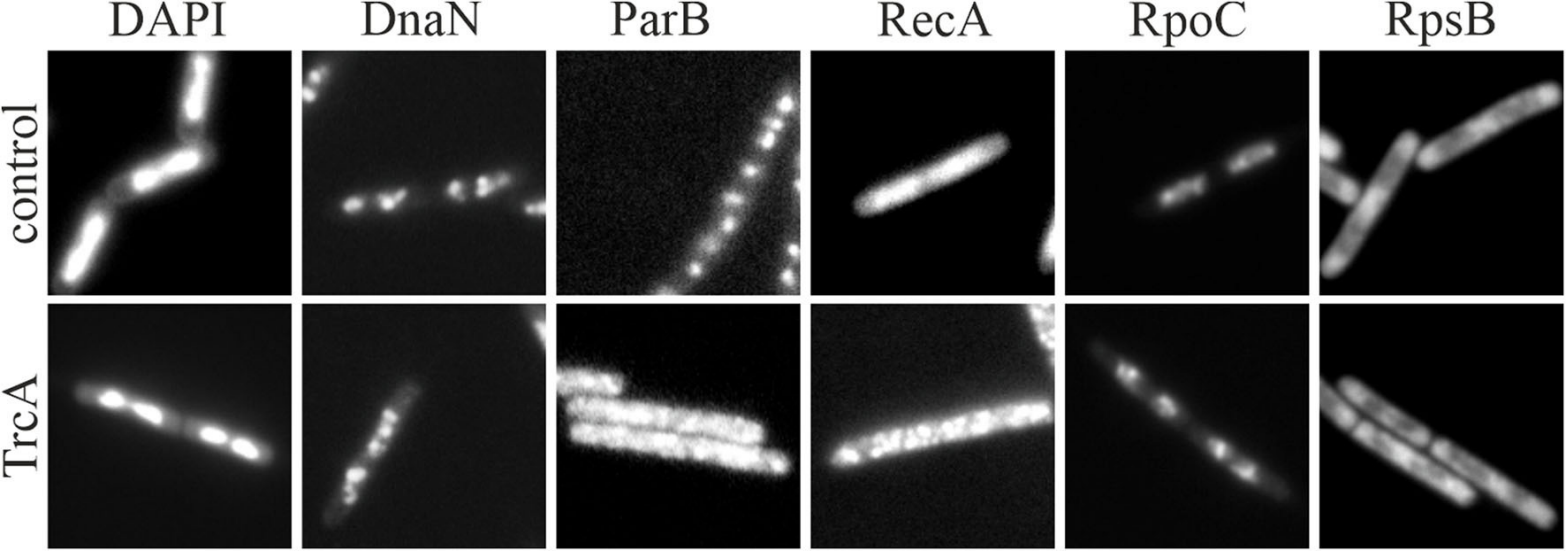
Microscopic assays for antibiotic effects on the nucleoid. Cells were treated with tyrocidine A, which has been described to bind to DNA (Ristow et al., 1975). DAPI is used as a DNA dye and shows clear chromosome compaction after treatment. DnaN and ParB are DNA-binding proteins and reporters for impaired replication. RecA is a reporter for DNA damage. RpoC is a reporter for impaired RNA synthesis. All these proteins showed a clear change in their localization pattern after exposure to tyrocidine A. RpsB is a ribosomal protein that is used as reporter for impaired protein synthesis, which was included as a negative control and showed no effect.

Bacterial cytological profiling can be employed for intracellular protein targets, since a disruption of their localization pattern is indicative of an interruption of the respective pathway (Figure 5). Several GFP fusions to intracellular targets have been used for this purpose (Table 4). Inhibition of one specific protein can also affect the functionality and localization of its interaction partners. While this makes BCP a great tool to study the effects of a compound in detail, it rarely identifies the target protein by itself and typically needs follow-up studies.

A more specific way of identifying protein targets is based on the detection of protein stability changes upon binding of an antibiotic ligand. Typically, binding of a ligand stabilizes the target protein and protects it from proteolytic degradation (Lomenick et al., 2009). This can be exploited for target identification by globally mapping protein stability with and without addition of the antibiotic compound. Proteins that display stability changes constitute potential targets. This method can, in principle, be performed on both cell extracts and whole cells and detection methods of the stability shift range from simple SDS-PAGE to advanced mass spectrometry techniques (Mateus et al., 2017).

Several other methods based on the principle of ligand-mediated protein stabilization have been developed. Thermoshift assays assess the resistance of proteins against denaturation by heat and can be easily done in a gradient PCR machine (Martinez Molina et al., 2013; Mateus et al., 2017; Webb et al., 2019). Drug affinity responsive target stability (DARTS) is a variant based on stability toward a specific protease (Lomenick et al., 2009). Stability of proteins from rates of oxidation (SPROX) measures the stability of proteins to the denaturing oxidative agent guanidinium hydrochloride (West et al., 2010; Strickland et al., 2013).

Another method to identify a potential protein target is target identification by chromatographic co-elution (TICC) (Chan et al., 2012). This method uses non-denaturing HPLC separation of cell lysate coupled with mass spectrometry. If a ligand antibiotic binds to a protein, its chromatographic retention time will change, and the protein will elute in a different fraction. Proteins can be identified by mass spectrometry and those appearing in different fractions in the treated and untreated samples are candidate target proteins (Chan et al., 2012). The current limitation with this method is that it has only been employed for soluble cytosolic proteins so far.

### Nucleic Acids

Nucleic acids and proteins that bind to them can be targeted by antibiotics and AMPs alike. Dyes to label DNA in living cells are well-established, most prominently DAPI and Hoechst DNA stains, but many more are available covering a range of fluorescent colors. These dyes allow observation of nucleoid morphology and can reveal defects like chromosome compaction or fragmentation (Wenzel et al., 2018a, 2019). Fluorescently labeled proteins such as the recombinase RecA and DNA polymerase can be used as reporters for DNA damage and inhibition of replication, respectively (Figure 5, Table 4).

The anucleate cell blue assay is a simple *E. coli* reporter assay that can be used to screen for compounds that target DNA partitioning. It is based on a plasmid-encoded beta-galactosidase that is regulated by a chromosome-encoded repressor. If DNA segregation is inhibited, anucleate cells are produced, which are stained blue due to derepression of the plasmid-encoded beta-galactosidase gene (Wachi et al., 1999). This assay can be used to identify topoisomerase inhibitors (Oyamada et al., 2006, 2007).

For transcription inhibition, RNA polymerase localization can be observed. However, since it is a DNA-binding protein, it will also be affected by DNA packing defects like chromosome compaction (Wenzel et al., 2018a). In contrast to DNA, there are not many RNA dyes available. Since research has mainly focused on visualizing specific transcripts, sequence-specific RNA labeling with RNA-binding proteins and hybridization techniques is well-established (van Gijtenbeek and Kok, 2017; Fei and Sharma, 2018). One general RNA dye is the green-fluorescent SYTO variant RNASelect, which is five times more selective for RNA over DNA and has been successfully employed for staining of RNA in live *E. coli* (Bakshi et al., 2014).

## Oxidative Stress

Disturbance of the respiratory chain can lead to the generation of reactive oxygen species, which damage cellular macromolecules including DNA, RNA, and membranes (Lee et al., 1998; Cabiscol et al., 2000; Yoon et al., 2002). Some antibiotics also generate reactive oxygen species by other means [e.g., nitrofurantoin (Tu and McCalla, 1975)].

One way to examine whether a compound causes oxidative damage is to monitor the oxidative stress response of bacteria. The reaction of bacteria like *B. subtilis* and *E. coli* to oxidative cell damage is well-characterized (Antelmann et al., 1997; Leichert et al., 2003; Leichert and Jakob, 2004). Transcriptomic and proteomic studies allowed delineating specific stress response proteins that can now be used as oxidative stress markers. Their induction can be monitored by reporter gene fusions or by stress response profiling. Proteins that are well-suited for this are for example catalase, superoxide dismutase, and thioredoxin (Leichert et al., 2003).

Several fluorescence dyes are available to detect reactive oxygen species, for example CellRox, which fluoresces red in presence of superoxide and singlet oxygen, the free radical sensor Oxyburst green, and 2′, 7′-dichlorodihydrofluorescein diacetate (DCFH-DA), which detects hydrogen peroxide and nitric oxide (Wenzel et al., 2013; Arakha et al., 2015; Surewaard and Kubes, 2017). All of these dyes penetrate bacterial cells and are oxidized to a fluorescent product by the respective reactive oxygen species. A broad palette of other dyes with different reactive oxygen species selectivity is commercially available, yet not all of them have been used in bacteria.

Both of these methods are not a direct proof of oxidative cell damage. Mass spectrometry however allows direct detection of oxidative damage to proteins by measuring cysteines in their thiol (reduced) and disulfide (oxidized) forms (Kozarova et al., 2007; Sethuraman et al., 2007). To this end, isotope-coded affinity tags (ICAT) can be used. Employing a selective labeling technique, OxICAT, reduced and oxidized cysteines can be labeled with a light or heavy isotope tag, respectively (Leichert et al., 2008). This method has been further refined to also detect nitrosative stress (NOxICAT) (Lindemann and Leichert, 2012).

## Conclusion

The pressure of antimicrobial resistance has prompted an urgent need for new antimicrobial compounds with novel mechanisms of action. Understanding these mechanisms is of high importance for two reasons. One the one hand, the mechanism must simply be known prior to clinical approval. On the other hand, it is of utmost importance to understand how successful antibiotics work in order to develop better compounds in the future. Antibiotics with single protein targets are inferior to compounds with complex or multiple targets in terms of resistance development (Brötz-Oesterhelt and Brunner, 2008) and antibiotic discovery is slowly shifting toward multifunctional compounds (Gajdács, 2019; Gray and Wenzel, 2020b). This poses a challenge for mode of action analysis, since these mechanisms are less well-understood and more difficult to diagnose. Moreover, it has become clear that antibiotic mechanisms may differ *in vitro* and in *in vivo* and that compounds may have additional targets in living cells (Müller et al., 2016b; Wenzel et al., 2019; Grein et al., 2020). This underlines the importance of *in vivo* mechanism of action studies. In this review, we have summarized a number of useful techniques for *in viv*o mode of action studies of antimicrobial compounds. We hope that it can serve as guide to researchers, who are less familiar with this type of experiments, to inspire new exciting mechanistic studies on living bacteria.

## Author Contributions

A-BS and MW: conceptualization, writing—original draft preparation, writing—review, editing, and visualization. All authors contributed to the article and approved the submitted version.

## Conflict of Interest

The authors declare that the research was conducted in the absence of any commercial or financial relationships that could be construed as a potential conflict of interest.
